# Detection of Water Contaminants by Organic Transistors as Gas Sensors in a Bottom-Gate/Bottom-Contact Cross-Linked Structure

**DOI:** 10.3390/s23187981

**Published:** 2023-09-20

**Authors:** José Enrique Eirez Izquierdo, Marco Roberto Cavallari, Dennis Cabrera García, José Diogo da Silva Oliveira, Vinicius Augusto Machado Nogueira, Guilherme de Souza Braga, Oswaldo Hideo Ando Junior, Alain A. Quivy, Ioannis Kymissis, Fernando Josepetti Fonseca

**Affiliations:** 1 Departamento de Engenharia de Sistemas Eletrônicos (PSI), Escola Politécnica da Universidade de São Paulo (EPUSP), São Paulo 05508-010, SP, Brazil; jeeizquierdo@usp.br (J.E.E.I.); cabreradennis20@usp.br (D.C.G.); josediogodasilva.oliveira@usp.br (J.D.d.S.O.); vinaoaugusto1998@usp.br (V.A.M.N.); guilherme.braga@inesctec.pt (G.d.S.B.); 2 School of Electrical and Computer Engineering, University of Campinas (Unicamp), Av. Albert Einstein 400, Campinas 13083-852, SP, Brazil; 3 Electrical Engineering Department, Columbia University, New York, NY 10027, USA; johnkym@ee.columbia.edu; 4 Institute for Systems and Computer Engineering, Technology and Science (INESC TEC), 4200-465 Porto, Portugal; 5 Research Group on Energy & Energy Sustainability (GPEnSE), Academic Unit of Cabo de Santo Agostinho (UACSA), Federal Rural University of Pernambuco (UFRPE), Cabo de Santo Agostinho 54518-430, PE, Brazil; oswaldo.ando@ufrpe.br; 6 Institute of Physics, University of São Paulo, São Paulo 05508-090, SP, Brazil; aquivy@if.usp.br

**Keywords:** organic thin-film transistors, organic semiconductors, PBTTT-C14, gas sensors, electronic nose, organic electronics

## Abstract

Detecting volatile organic compounds is a fundamental step in water quality analysis. Methylisoborneol (MIB) provides a lousy odor to water, whereas geosmin (GEO) is responsible for its sour taste. A widely-used technique for their detection is gas-phase chromatography. On the other hand, an electronic nose from organic thin-film transistors is a cheaper and faster alternative. Poly(2,5-bis(3-tetradecyl-thiophen-2-yl)thieno[3,2-b]thiophene) (PBTTT-C14) features semiconducting properties suitable for organic electronics. However, in order to expose the active layer in a bottom-gate transistor structure with photolithographically patterned electrodes, a cross-linked dielectric such as poly(4-vinyl phenol) (PVP) is necessary. In this work, the cross-linking was demonstrated using FTIR and Raman spectroscopies, as well as high-k capacitors with a dielectric constant of 5.3. The presence of enhanced crystallinity with terrace formation in the semiconducting film was confirmed with UV-visible spectrophotometry, atomic force microscopy, and X-ray diffraction. Finally, for the first time, a PBTTT-C14 transistor on cross-linked PVP was shown to respond to isoborneol with a sensitivity of up to 6% change in mobility per ppm. Due to its similarity to MIB, a system comprising these sensors must be investigated in the future as a tool for sanitation companies in real-time water quality monitoring.

## 1. Introduction

**Figure 1:**
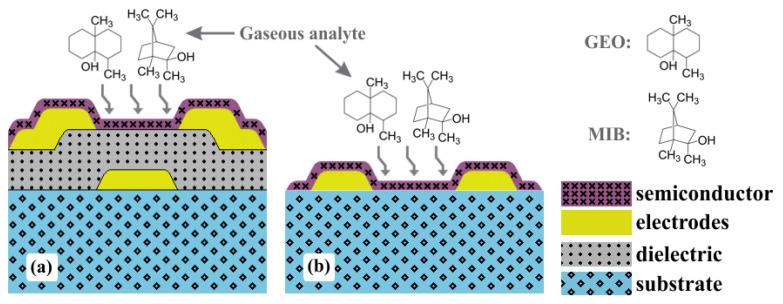
Schematic structure of gas sensor devices from (**a**) bottom-gate/bottom-contact (BGBC) OTFTs, and (**b**) chemical sensors.

Organic electronics, compared with conventional silicon-based microelectronics, offers a myriad of semiconducting and dielectric materials, as well as low-cost large-area deposition techniques at room temperature and atmospheric pressure. Organic thin-film transistors (OTFTs) are one of the fundamental building blocks of electronic circuits. Despite the fact that charge carrier mobility (μ) in organic semiconductors is orders of magnitude lower than that in silicon, many recently synthesized materials have already overcome amorphous silicon performance. Additionally, OTFTs can be fabricated not only on hard substrates such as glass and silicon [1,2], but also on flexible polyimide (PI) [3], poly(ethylene terephthalate) (PET) [4], and poly(ethylene naphthalate) (PEN) [5]. The higher mobility, enhanced mechanical flexibility, and biocompatibility of organic materials opens up the way to unknown applications for humans. Among them, it paves the way for gas and liquid sensors in general-purpose electronic noses and tongues in the food and beverage industries, medical diagnosis, and water quality assessment [6,7,8,9]. An OTFT as a gas sensor requires a bottom-gate structure in order to expose the active layer. The ability to chemically resist source and drain patterning and semiconductor deposition usually demands orthogonal solvents or a cross-linked dielectric film. The structure shown in Figure 1a with fully-patterned electrodes is an important step towards miniaturization and integration with signal amplification and processing circuits [6,8]. Compared with the chemical sensors shown in Figure 1b, OTFTs have additional interfaces for enhanced sensing responses as well as additional electrical parameters for analyte classification. Whereas chemical sensor performance is often monitored through the resistive component of the electrical impedance, transistors minimally provide drain current ($I_{D}$), charge carrier mobility, and threshold voltage ($V_{T}$) [6,10].

The evaluation of water’s bad taste and odor is often linked to the detection of geosmin (GEO) and methylisoborneol (MIB), respectively [11]. These tainting compounds are a recurring problem for water utilities around the world and present a trend of worsening with time due to an increase in nutrient inputs in water reservoirs combined with elevated water surface temperatures [12,13]. Also, the presence of those off-flavors in drinking water is one of the major sources of complaints for water companies, causing a negative impact on a company’s reputation [14,15]. In 2022, Wu et al. [16] used a headspace solid-phase microextraction (HSPME) method coupled with gas chromatography-mass spectrometry (GC-MS) to investigate contamination in a subtropical drinking water reservoir in southeast China. Despite detection limits lower than 1 ng/L, the observed peak concentrations were 87.22 ng/L and 7.67 ng/L for contamination by MIB and GEO, respectively [16]. Similarly, Xu et al. [17] used GC-MS to quantify a decrease in MIB concentration following pre-ozonation coupled with a post-peroxone (O${}_{3}$/H${}_{2}$O${}_{2}$) process to remove filamentous cyanobacteria. MIB concentrations of ca. 60–70 ng/L in Chengbei Reservoir, China, were reduced to 3.8 ng/L after treatment [17]. GC-MS was also employed to quantify contaminants in a recirculated aquaculture system to raise Japanese seabass in the presence of actinobacteria and cyanobacteria [18]. In this simulated environment, Lu et al. [18] demonstrated GEO and MIB levels of 169 ng/L and 45 ng/L, respectively.

The detection of similar contamination levels was already demonstrated by Braga et al. [14] with chemical sensors (CS) from conducting polymers such as polyaniline (PANI) and polyallylamine hydrochloride (PAH). On the other hand, the application of a field-effect transistor platform with organic compounds is rather new. As mentioned previously, it could decrease measurement complexity, processing, cost, and time compared with GC-MS, while offering the possibility of signal amplification and integration compared with CS. In 2015, Son et al. developed a bioelectronic nose from single-walled carbon nanotubes (swCNTs) to selectively detect MIB and GEO at concentrations as low as 10 ng/L [19]. In very recent work, Park et al. [20] used aptamer-conjugated graphene to mimic the olfactory nose and detect GEO in a 0.01 nM to 1 µM concentration interval [20].

Alternatively, polymer semiconductors as polythiophene derivative poly (2,5-bis (3-tetradecyl-thiophen-2-yl) thieno[3,2-b] thiophene) (PBTTT-C14) have also been shown to be suitable for gas-sensing applications [21,22,23]. Depending on the processing parameters, the semiconducting film can be either amorphous or form terraces and nodules, which directly relates to the electrical performance [24]. Sahu et al. [22] demonstrated a mobility variation of ca. 19% at 20 ppm ammonia concentration in a bottom-gate/top-contact (BGTC) structure with electrodes evaporated through a shadow mask on Si/SiO${}_{2}$. In a similar structure, Singh et al. [23] were able to detect ammonia at 2 ppm. PBTTT-C14 also has the potential for alcohol detection [23,25]. Dumitru et al. demonstrated ca. 5% *I*${}_{D}$ variation under 39 ppm of 1-butanol. Despite an alternative structure with an electrolyte gate of poly(4-styrene sulfonic acid) (PSSH), once again, top contacts were evaporated through a shadow mask on a Si/SiO${}_{2}$ substrate [25]. Poly(4-vinyl phenol) (PVP), on the other hand, is a polymer dielectric fitted to integrate BGBC-OTFTs in gas-sensing applications [26,27]. Depending on the presence of cross-linking, it is possible to withstand lithography to form bottom contacts [28]. However, researchers avoid performing photolithography on top of organic dielectric materials [1,29]. For instance, Chou et al. chose to thermally evaporate silver source and drain electrodes through a shadow mask on top of the polymer semiconductor [1]. Its high mobility (ca. 1 cm${}^{2}$/Vs) in TFTs is not only related to the semiconductor deposition technique, but also to the choice for a BGTC structure eliminating photolithography. In general, BGBC-OTFTs over glass are not commonly used in sensor applications [30]. In fact, even nowadays, a Si/SiO${}_{2}$ substrate is still widely adopted for performing source and drain photolithography in PBTTT-C14 TFTs as gas sensors [31,32].

In this context, this work focused on the development of OTFTs from PBTTT-C14 on top of cross-linked PVP with bottom electrodes patterned by photolithography as a platform for water analysis. It contributes to developing e-noses for the food, beverage, agricultural, and pharmaceutical industries. Isoborneol (IB) was chosen as the target molecule due to its chemical similarity with MIB and its wide availability. It was dissolved in methanol due to its poor solubility in water. Since this field of research is closely related to chemistry and biology, it has enormous commercial potential. In addition, this research implements the following goals of UNESCO’s roadmap for sustainable development: (i) 3—good health and well-being; (ii) 6—clean water and sanitation; and (iii) 14—life below water.

## 2. Materials and Methods

### 2.1. Materials

**Figure 2:**
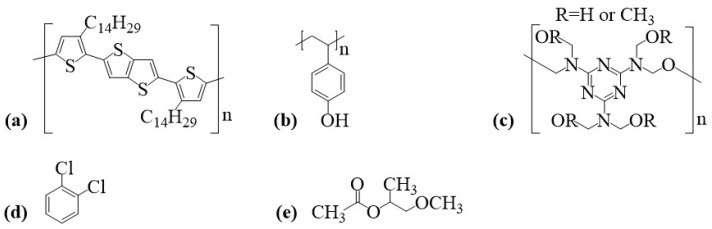
Chemical structure of compounds for polymer solutions: (**a**) PBTTT-C14, (**b**) PVP, (**c**) PMF, (**d**) DCB, and (**e**) PGMEA.

Samples were processed over BK7 glass (2.5 cm ×2.5cm×0.1 cm, Opto Eletrônica S/A, São Carlos, SP, Brazil) and low-doped Si (p-type, <100>, 3 in diameter, 10–20 Ω·cm resistivity, 14–16 mils thick, prime grade) substrates. PBTTT-C14 (Mn=50 kg/mol, PD=3) semiconducting p-type polymer was dissolved in 1,2-dichlorobenzene (DCB: Mn=147 kg/mol, HPLC, purity/P = 99%). PVP (MW=25 kg/mol) dielectric polymer was cross-linked with poly(melamine-co-formaldehyde) methylated (PMF: Mn∼432, 84 wt.% in 1-butanol, number: 418560) and dissolved in propylene glycol methyl ether acetate (PGMEA: Mn=132.16 g/mol, HPLC, P=99.5%). Polymers and solvents were purchased from Sigma-Aldrich (St. Louis, MO, USA) and used without further purification. Chemical structures of compounds for device fabrication are given in Figure 2.

**Figure 3:**
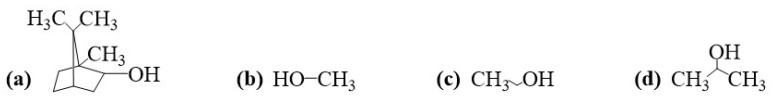
Chemical structure of compounds for gas-sensing experiments: (**a**) IB, (**b**) MET, (**c**) ETA, and (**d**) IPA.

Liquid analyte samples were prepared from isoborneol powder (IB: MW=154.25 g/mol, P=93%, Acros Organics, Thermo Fisher Scientific, Waltham, MA, USA), dissolved in methanol (MET: ρ=0.79 g/mL at 25 ${}^{\circ }$C, P=99%, MW=32.04 g/mol, P=98%, Casa Americana, São Paulo, SP, Brazil), and further diluted in water to achieve lower concentrations. Pure methanol, ethanol (ETA: ρ=0.789 g/mL at 20 ${}^{\circ }$C, P≥99.5%, Sigma Aldrich), isopropyl alcohol (IPA: ρ=0.785 g/mL at 25 ${}^{\circ }$C, Sigma Aldrich), and ultrapure water (Millipore sterile filter 0.22 μm, Milli-Q, 18 MΩ·cm) were also investigated in gas sensors for comparison purposes. Chemical structures of compounds for gas-sensing experiments are shown in Figure 3. All molecules were drawn in ChemDraw 19.1 software.

### 2.2. Device Processing

Organic thin films were investigated both on silicon (only for FTIR experiments) and glass. Substrates were first cleaned in sequential 10 min baths in acetone (ACE), deionized (DI) water, and isopropyl alcohol, followed by drying under N${}_{2}$ flow. Dielectric PVP:PMF at 1:0 and 1:5 mass ratio was dissolved in PGMEA at 6.3, 11, and 16 wt.% and agitated for 24 h before filtration. These solutions were spun at 600–5000 rpm for 60 s with 5 s acceleration/deceleration steps. Thin films were then dried on a hot plate at 100 ${}^{\circ }$C for 10 min and crosslinked at 175 ${}^{\circ }$C for 120 min. These processes were performed in ambient conditions. Semiconductor PBTTT-C14 was dissolved in DCB from 2 to 10 mg/mL and heated at 100 ${}^{\circ }$C for 1 h (more details are given in the Supplementary Materials). The solution was either drop cast (only for FTIR experiments) or spun at 600–3000 rpm for 60 s with 2.5 s acceleration/deceleration steps. Thin film was then dried according to one of the following conditions:

- (A) room temperature and 1 atm in a fume hood overnight;
- (EA) room temperature and 0.2 bar in a vacuum oven for 20 min;
- (H80) 80 ${}^{\circ }$C and 1 atm on a hot plate in a fume hood for 20 min;
- (E80) 80 ${}^{\circ }$C and 0.2 bar in a vacuum oven for 20 min;
- (H150) 150 ${}^{\circ }$C and 1 atm on a hot plate in a fume hood for 20 min;
- (E150) 150 ${}^{\circ }$C and 0.2 bar in a vacuum oven for 20 min;
- (H150N) 150 ${}^{\circ }$C and 1 atm on a hot plate inside an N${}_{2}$-filled glovebox (i.e., O${}_{2}$ and moisture levels under 10 ppm) for 20 min.

**Figure 4:**
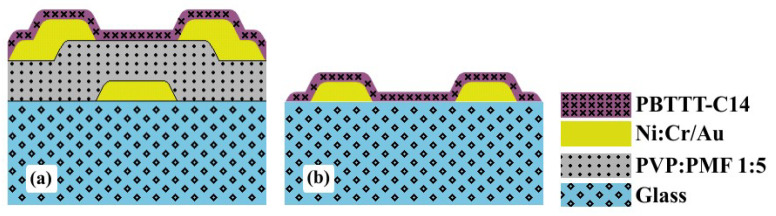
Thin-film structure for: (**a**) transistors and (**b**) chemical sensors over glass (not in scale).

Transistors were BGBC devices with fully-patterned electrodes on glass (see Figure 4a). The gate, source, and drain were deposited in a DC sputtering BAE 370 (BALZERS, Switzerland). Ni:Cr adhesion layer (5±2 nm thick) was deposited at 0.8 Å s^−1^ and 90 ${}^{\circ }$C. Au (95±4 nm thick) was deposited at 1.7 Å s^−1^ and 90 ${}^{\circ }$C. Photolithography was performed in a Karl Suss KG, GmbH & Co. (SUSS MicroTec, Garching, Germany) mask aligner in a cleanroom environment. The photomasks (see Figure S1 in the Supplementary Materials) featured (i) 8 OTFTs with interdigitated source and drain electrodes (50 pairs of 10 μm wide and 3000 μm long digits; channel length −L− of 4, 6, 8, 10, 12, 14, 16, and 20 μm with a width −W− of 300,000 μm); and (ii) 8 capacitors (area −A− of 500 μm × 500 μm, 1000 μm ×1000 μm, and 2000 μm × 2000 μm). Organic films were processed as described in the previous section, except for the following particularities: (i) PVP required PMF at 1:5 mass ratio dissolved at 16 and 25 wt.% in PGMEA, and spun at 2000 rpm; and (ii) PBTTT-C14 was dissolved in DCB at 6 and 10 mg/mL, spun at 600 rpm, and dried at H150N.

Chemical sensors, as shown in Figure 4b and listed in Table 1, were processed just for the sake of gas-sensing performance comparison. Fabrication parameters were the same as those for OTFTs, except for the absence of a gate electrode and dielectric. In this case, the photomask was composed of 50 pairs of 10 μm wide and 5000 μm long digits with 10 μm separation (see Figure S2 in the Supplementary Materials). Note, however, that the W/L ratio was approx. 5/3 higher for CS compared with OTFTs.

In this work, 18 samples (2.5 cm ×2.5 cm ×0.1 cm) containing TFTs (also interrogated as MIS capacitors) and 144 parallel-plate MIM capacitors were fabricated. A total of 20 OTFTs, 17 MIS, and 24 MIM capacitors were characterized. The low yield is mostly related to optimizing the dielectric cross-linking to withstand photolithography and semiconductor deposition. In gas-sensing experiments, 8 OTFTs from 4 different samples were investigated. An amount of 35 chemical sensors were fabricated (1.25 cm ×2.5 cm ×0.1 cm) and measured as gas sensors.

### 2.3. Morphological and Surface Characterization

Thickness ($x_{s}$) and RMS roughness (Rq) were investigated using atomic force microscopy (AFM) on a NanoScope^®^ V with ScanAsyst (BRUKER, Santa Barbara, CA, USA) with NANOWORLD microtips (70 kHz resonance frequency and 0.4 N/m spring constant). UV-visible absorption spectra were acquired in a UV-1650 PC spectrophotometer (SHIMADZU, Kyoto, Japan) for a wavelength (λ) range of 300–1100 nm. Spectra were compared according to the wavelength at maximum absorbance ($\lambda_{cp}$) and at a secondary local peak ($\lambda_{sh}$). These parameters were calculated using the first derivative method given in [33]. Fourier transform infrared (FTIR) spectroscopy was performed in a QS-300 FTS-40 (BIO-RAD, Hercules, CA, USA) at ambient conditions, wavenumber of 400–4000 cm${}^{-1}$ with 8 cm${}^{-1}$ resolution, 5 kHz frequency, and operated with Win-IR Pro software. Raman spectra were obtained in a Confocal Raman Microscope Alpha300 R (WITec, Ulm, Germany) at ambient conditions, wavenumber of 0–3794 cm${}^{-1}$ with 5 cm${}^{-1}$ resolution, laser λ of 532 nm with a maximum power of 45 mW, and operated with Control FIVE 5.1 software. X-ray diffraction (XRD) was performed in an XRD 6000 (SHIMADZU, Japan) from a Cu tube, 2∗θ from 3 to 20${}^{\circ }$, scan rate of 1 s/step, scan speed of 2${}^{\circ }$/min, divergence slit of 1${}^{\circ }$, scatter slit of 1${}^{\circ }$, and receiving slit of 0.3 mm. Crystallographic plane separation (*d*) was determined according to [34]:

(1) $$d=\frac{n*\lambda }{2*sin\theta },$$

where n=1, since the highest intensity peak is used in the calculation, λ=1.54184Å from a copper X-ray source, and θ is the angle at the peak in radians. Data treatment and plotting were performed in Origin^©^ 2019 software. The calculation of each parameter given in this section was based on at least three different measurements. The approximate total of samples used in each optical, morphological, and structural study was 119 for UV-vis, 74 for AFM, 16 for Raman, 11 for FTIR, and 14 for XRD.

### 2.4. Electrical Characterization

#### 2.4.1. Capacitors

The impedance (*Z*) as a function of frequency (*f*) data from Metal–Insulator–Metal (MIM) capacitors were acquired in Autolab^®^ PGSTAT302N with an FRA32M module. The device was interrogated with a 0.5 Vpp sinusoidal signal. The dielectric constant (*k*), assuming an ideal parallel-plate capacitor, was calculated from the following equation [35]:

(2) $$Z=\frac{1}{2*\pi *f*C_{ins}}=\frac{x_{ins}}{2*\pi *f*k*\epsilon_{0}*A},$$

where $C_{ins}$ is the dielectric capacitance, $\epsilon_{0}$ the vacuum permittivity, *A* the area of the plates, and $x_{ins}$ their separation. On the other hand, Metal–Insulator–Semiconductor (MIS) capacitors were measured on a B1500 Semiconductor Parameter Analyzer (KEYSIGHT, Santa Rosa, CA, USA) with C−V module and short-circuited S/D electrodes. The DC bias voltage sweep applied to the gate ($V_{GS}$) ranged from −25 to 25 V, i.e., from hole accumulation to depletion. For negative $V_{GS}$, mobile carriers accumulated in the channel, thus increasing its conductivity and capacitance and reaching its maximum ($C_{ins}$). Conversely, the channel was depleted for more positive $V_{GS}$ because positive charge carriers moved away from the dielectric/semiconductor interface. Therefore, the total capacitance was expected to reach its minimum and equaled the series association of the depleted semiconductor capacitance ($C_{s}$) with $C_{ins}$.

#### 2.4.2. Transistors

The electrical characterization of OTFTs was initially performed inside a N${}_{2}$-filled glovebox with a KEYSIGHT B1500A and in the dark. The acquired measurements were (i) $I_{D}$ versus $V_{GS}$ transfer curve for $V_{GS}$ from 10 to −10 V with a −0.05 V step and $V_{DS}$ from 0 to −10 V, with a −1 V step; and (ii) $I_{D}$ versus $V_{DS}$ output curve for $V_{DS}$ from 0 to −10 V with a 0.05 V step and $V_{GS}$ from 4 to −10 V, with a −2 V step. In order to avoid bias-stressing the device [36,37], once in the gas chamber, just the $I_{D}$ versus $V_{GS}$ transfer curve was acquired, being limited to a $V_{GS}$ scan from 1 to −1 V with a −0.05 V step at $V_{DS}=-1$ V.

Hole mobility in the triode region ($\mu_{p,tri}$) was extracted from the maximum of transconductance ($g_{m,max}$) as a function of $V_{GS}$ for $V_{DS}=-1$ V and according to the following [38,39]:

(3) $$\mu_{p,tri}=\frac{g_{m,max}}{\frac{C_{ins}}{A}*\frac{W}{L}*V_{DS}}.$$

Hole mobility in the saturation region ($\mu_{p,sat}$) was extracted from the slope of the linear fit of the square root of $I_{D}$ as a function of $V_{GS}$ for $V_{DS}=-10$ V and according to the following [39]:

(4) $$\mu_{p,sat}=\frac{2*L*{\left(\frac{\partial \sqrt{I_{D}}}{\partial V_{GS}}\right)}^{2}}{W*\frac{C_{ins}}{A}}.$$

The threshold voltage was determined from the minimum of the second derivative of $I_{D}$ as a function of $V_{GS}$ for $V_{DS}=-1$ V [40]. On currents ($I_{ON}$) were extracted from $I_{D}$ at $V_{GS}=-10$ V for $V_{DS}=-10$ V in saturation regime. As for the off currents ($I_{OFF}$), they were extracted at $V_{GS}=10$ V for $V_{DS}=-10$ V with the OTFT in the cut-off regime.

The subthreshold slope (SS) was obtained from $\log_{10}\left|I_{D}\right|$ versus $V_{DS}$ plots for $V_{DS}=-1$ V and according to [38]:

(5) $$SS=\frac{\partial V_{GS}}{\partial \log_{10}\left|I_{D}\right|}.$$

Since gas-sensing data were extracted in a much shorter $V_{GS}$ interval, a linear fit was performed to calculate $V_{T}$ [38,39]. For the same reason, $I_{ON}$ was determined to be the average current in that same $V_{GS}$ scan. More details on parameter extraction from OTFT data can be found in [10,36,37].

### 2.5. Gas-Sensing Measurements

Liquid analyte samples were prepared from IPA (*c* = 10–600 ppm), ETA (*c* = 10–800 ppm), MET (*c* = 10–1000 ppm), and IB (*c* = 10–800 ppm) in ultrapure water. Due to IB’s poor solubility in water, it was initially dissolved in MET at 150 mg/mL to be later diluted in ultrapure water. A total of 140 liquid analyte samples were prepared for 7 experiments at 5 concentrations of 4 analytes. Each concentration was determined using the following expression for a dilution [41]:

(6) $$V_{1}=\frac{c_{2}*V_{2}}{c_{1}},$$

where $c_{1}$ (ppm) is the initial concentration of the analyte, $V_{1}$ (L) the initial pipetted volume of the analyte, $c_{2}$ (ppm) the final analyte concentration, and $V_{2}$ (L) the final volume of the solution. The concentration $c_{1}$ was calculated according to [42]:

(7) $$c_{1}=\frac{P}{100}*\rho *10^{6}.$$

Analyte solutions were placed inside a bubbler and carried by N${}_{2}$ to two different chambers simultaneously, i.e., one with CS and a separate one with OTFTs (see Figure S3 in the Supplementary Materials). This was identified as a wet N${}_{2}$ flow, with relative humidity (RH) approaching 100%. Further dilutions to adjust relative humidity, as well as the reference atmosphere with pure N${}_{2}$, were due to a secondary dry N${}_{2}$ flow (RH ≈ 0%). Both flows were controlled by rotameters, pressure regulators, and valves, with a temperature and humidity meter along the outlet (HTR170, Instrutherm, São Paulo, SP, Brazil). The total flow was kept constant at 2.4 L/min. The chamber where the transistors were measured had a volume of 2.5 cm ×2.5 cm ×1 cm, which led to a purge time of less than 0.310 s. Note that, considering the chamber for CS was larger than the one for OTFTs [43], OTFTs’ responses were expected to stabilize in a shorter time.

A parallel RC circuit was used as a model for chemical sensors. Resistance (*R*) and capacitance (*C*) data were acquired continuously with a KEYSIGHT E4980AL LCR Meter at $V_{RMS}=0.25$ V and a frequency of 1 kHz (see Figure S4 in the Supplementary Materials). OTFT data were taken once a plateau had been observed from CS data. The $V_{GS}$ sweep was performed between −1 and 1 V with a 20 mV step for $V_{DS}=-0.5$ V. After each data point, sensors were reset under pure N${}_{2}$ for 30–60 min. All measurements were performed at ambient temperature (ca. 25 ${}^{\circ }$C) and a maximum pressure of 1.36 atm. Communication between the OTFT characterization box (see Figure S5 in the Supplementary Materials) and the KEYSIGHT B1500 was performed with triaxial cables. The user could choose which transistor to investigate by using three switches, i.e., one for the gate, one for the source, and one for the drain. More information on the CS measurement system can be found in [43].

Data matrices were generated after preprocessing signals, as described before. Columns represented the variation in gas sensors’ electrical parameters at RH=15% from 0 to *c* ppm of a certain analyte with respect to the variation from 0 to 15%RH at zero analyte concentration ($\Delta X/X_{0}$ at *c* ppm $=\left(X_{cppm,15\%RH}-X_{0ppm,15\%RH}\right)/\left(X_{0ppm,15\%RH}-X_{0ppm,0\%RH}\right)$, where X=μ, $V_{T}$, $I_{ON}$, *R*, or *C* and *c* is the concentration in ppm of MET, ETA, IPA, or IB) [10]. Rows indicated sensor response for each concentration of measured analytes. Matrices underwent principal component analysis (PCA), a statistical method for analyzing data matrices with multiple variables. PCA correlated data collected by the gas sensors, showing sample similarity. It identified sensor–sample correlations. PCA reduced the initial data matrix to a smaller matrix (*Z*) using variance and covariance matrix (*S*). Eigenvalues and eigenvector matrix (*U*) were extracted from *S*. Eigenvalues represented the variance in Principal Components (PC) as a percentage. The scalar product of eigenvectors indicated the angle between new and original coordinate systems. Finally, *Z* was obtained by multiplying the initial data matrix by *U*. MATLAB R2015a’s Statistical Toolbox (MATHWORKS, Natick, MA, USA) was used to implement the computational routine. More details on the aforementioned statistical analysis can be found in [44].

## 3. Results and Discussion

### 3.1. Morphological and Surface Studies of PBTTT-C14 Thin Films

**Figure 5:**
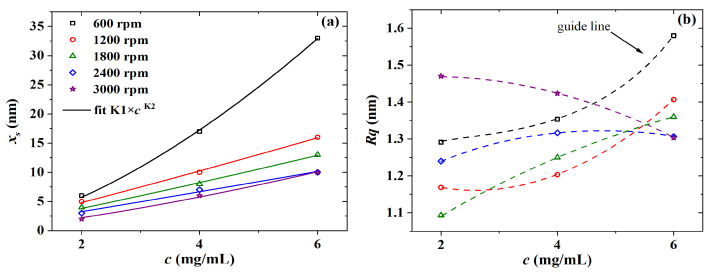
(**a**) Thickness and (**b**) roughness versus the concentration of PBTTT-C14 in DCB for films spun over glass and dried overnight under ambient conditions. Continuous lines represent fitted data, whereas dashed lines are just a guide for the eye.

Initially, PBTTT-C14 thin-film formation was investigated via AFM and evaluated through thickness and roughness determination. As shown in Figure 5a, there is a nonlinear direct relation between film thickness and solution concentration, which can be modeled by $x_{s}=K_{1}\times c^{K_{2}}$, where $K_{1}$ [nm·mL/mg] and $K_{2}$ are fitting constants [45]. The exponent $K_{2}$ is a system-specific parameter, which is expected to approach unity but is generally larger than one [46,47]. In this work, this deviation was even higher at lower spinning speeds. For instance, the $K_{2}$ values of films spun at 600, 1200, and 1800 rpm were 1.6, 1.1, and 1.1, respectively (see Table 2). In addition, the thickness of films from 2 mg/mL solutions barely depended on spinning frequency. A wider range of thickness was obtained only at higher concentrations, such as 10–33 nm of films from a 6 mg/mL solution. Roughness data are given in Figure 5b. Rq was lower than 1.5 nm for films spun from 2 to 4 mg/mL solutions. However, the trend observed in Rq as a function of *c* for films spun at 3000 rpm, compared with the ones at lower spinning frequencies, is a sign of malformation. On the other hand, at higher concentrations, such as 6 mg/mL, there was a clear increase in Rq from 1.3 to 1.6 nm alongside an increase in film thickness from 10 to 33 nm, respectively. These results agree well with previous reports [6,46,48] and point to 6 mg/mL as a lower limit for solution concentration in fabricating PBTTT-C14 TFTs as gas sensors.

**Figure 6:**
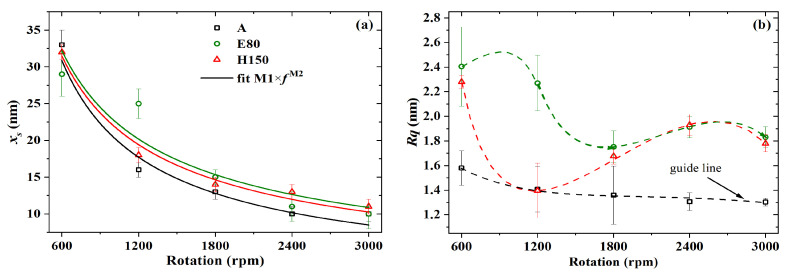
(**a**) Thickness and (**b**) roughness versus spinning frequency of PBTTT-C14 films spun from 6 mg/mL solution in DCB over glass and annealed under three different temperatures. Continuous lines represent fitted data, whereas dashed lines are just a guide for the eye.

The thickness and roughness of PBTTT-C14 films from 6 mg/mL solutions in DCB were also studied as a function of spinning frequency. In this case, however, three different annealing temperatures were applied to the films. As shown in Figure 6a, there is an inverse non-linear relation between thickness and deposition frequency. Upon fitting according to $x_{s}=M_{1}^{M_{2}}$ [45], it was found that $M_{2}$ lies, on average, between −0.7 and −0.8 (see Table 3). The exponent $M_{2}$, originally expected to be close to 0.5, has been shown for polymer films to lie between 0.5 and 1.0 [49]. Yimsiri and Mackley [50] showed that the less volatile the solvent (e.g., DCB), the closer to 1.0$M_{2}$ gets. In this work, higher speeds such as 1800 rpm did not seem to produce a noticeable change in film thickness. In addition, the decrease in Rq at higher spinning frequencies is once again observed in Figure 6b. This trend, however, was more distinguishable for films treated at room temperature. Thermal annealing at temperatures higher than or equal to 80 ${}^{\circ }$C can form films with an Rq as high as 2.4 nm at 600 rpm. Rougher films spun at lower frequencies and treated at higher temperatures may be desirable for sensing applications, since they facilitate gaseous analyte permeation through the film while providing a higher surface-to-volume ratio [21,51,52].

Table 4 provides a summary of the thickness and RMS roughness of PBTTT-C14 spun at 600 rpm from 6 mg/mL solutions. Depending on the annealing process, the thickness varied from 25 to 41 nm, whereas Rq ranged from 1.6 to 3.2 nm. As mentioned earlier, these values are acceptable for gas-sensing applications [10]. The thickest value was obtained in a vacuum oven at 150 ${}^{\circ }$C (E150). It is believed that, in this case, the film dried first on the outside and only later on the inside, leaving empty spaces to be filled up with air later. Therefore, this bloated film is believed to, in fact, be porous on the inside [10,53]. Another explanation lies in the packing of polymer molecules in the film. According to Cho et al. [54], a thicker film can be a consequence of the side chains being in contact with the substrate, i.e., edge-on positioning instead of face-on. This could favor molecular packing in the lamellar direction during solvent evaporation [54]. Other than that, there seems to be no direct correlation between thickness and annealing parameters. As observed in Figure 6b, the roughest film was dried in a vacuum oven. However, there was no clear trend of Rq with respect to drying environmental conditions.

**Figure 7:**
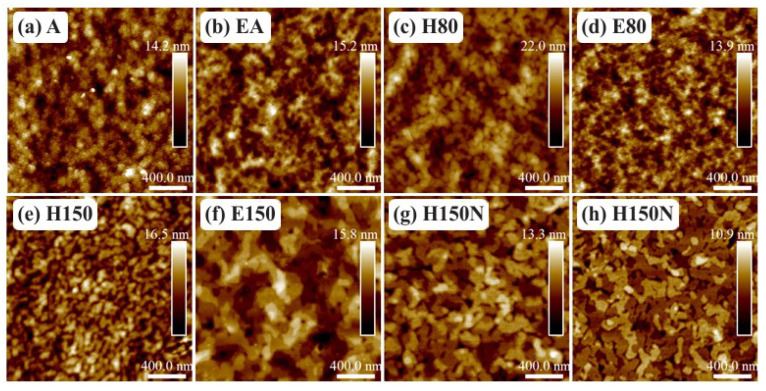
AFM micrographs of PBTTT-C14 films spun from a 6 mg/mL solution in DCB at 600 rpm/60 s over glass and annealed according to: (**a**) A, (**b**) EA, (**c**) H80, (**d**) E80, (**e**) H150, (**f**) E150, and (**g**) H150N. (**h**) Film spun from an 8 mg/mL concentrated solution and treated according to H150N.

The surface of the films mentioned in Table 4 is shown in Figure 7. According to previous work [31,55,56], nodules and terraces are present on the surface of crystalline PBTTT-C14. Based on that, films treated at room temperature (A and EA) or in a vacuum oven at 80 ${}^{\circ }$C (E80) are amorphous [56]. At higher temperatures, such as 150 ${}^{\circ }$C, but in the presence of O${}_{2}$ and moisture (H150), terrace formation is impaired. The presence of terraces in both films from 6 and 8 mg/mL solutions in DCB spun in an inert atmosphere and treated at 150 ${}^{\circ }$C (H150N) demonstrates that environmental conditions and temperature are more important than solution concentration [55]. In addition, filtering is not an issue at these concentration levels. Note that well-ordered terraces were also present after the treatment at 150 ${}^{\circ }$C in a vacuum oven (E150). Finally, in between these two extremes, drying on a hot plate at 80 ${}^{\circ }$C (H80) led to the formation of nodules [56]. The presence of terraces significantly enhances the electrical performance of the transistors [24,57].

**Figure 8:**
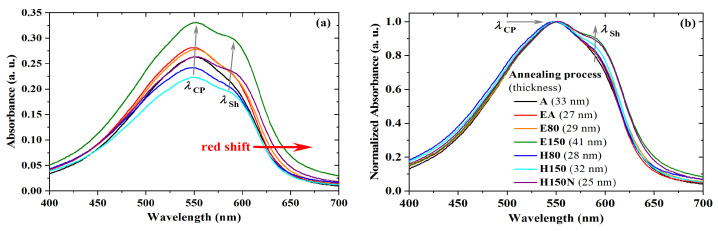
UV-vis (**a**) absorbance and (**b**) normalized absorbance of PBTTT-C14 films spun at 600 rpm/60 s from 6 mg/mL solution in DCB over glass for different annealing processes.

The absorbance in the UV-visible range was also investigated for different thermal treatments of PBTTT-C14 films. It can be seen as an indirect way to evaluate film crystallinity as well as semiconducting molecule structural degradation [56,58,59,60]. As shown in Figure 8a, there is a redshift of $\lambda_{cp}$ with an increase in treatment temperature. According to Jung et al. [60], a red shift of the spectra is an indirect sign of enhanced crystallinity of the semiconducting film. In this work, it happened only for films treated at 150 ${}^{\circ }$C in an atmosphere with reduced oxygen and moisture content. Accordingly, a $\lambda_{cp}$ of 552 nm is a sign of well-ordered backbone packing, which was observed both under low vacuum and inside the glovebox. In agreement with previous results, Wang et al. [56] stated that a decrease in thin-film drying temperature produces a blue shift in the absorbance spectra. In this case, however, it is noted that also the presence of O${}_{2}$ and H${}_{2}$O in the atmosphere tends to form amorphous films. That was observed at room temperature (A: $\lambda_{cp}=551$ nm), at 80 ${}^{\circ }$C (H80: $\lambda_{cp}=549$ nm), and even at 150 ${}^{\circ }$C (H150: $\lambda_{cp}=550$ nm). These trends are better-noticed after normalization, as shown in Figure 8b. In this case, a secondary peak ($\lambda_{sh}$) becomes distinguishable for films treated at 150 ${}^{\circ }$C, being barely perceptible for films dried in a vacuum oven at 80 ${}^{\circ }$C. Previously described in other work [61,62], this shoulder is usually at 585–592 nm and, according to Lee et al. [24], provides evidence of strong intermolecular coupling.

**Figure 9:**
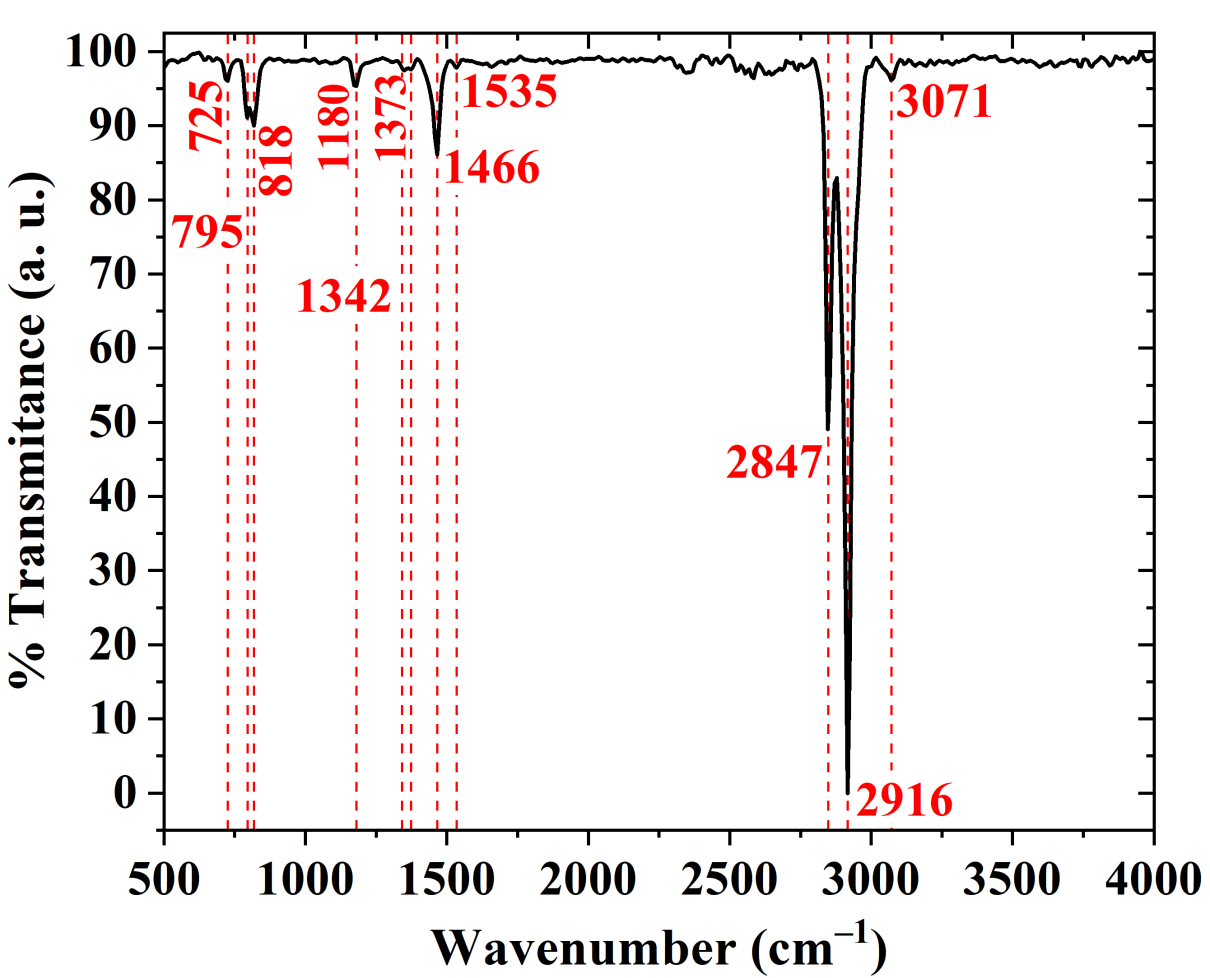
FTIR transmittance of a drop-cast PBTTT-C14 film from a 10 mg/mL solution in DCB over Si and treated according to H150.

An FTIR spectrum of a PBTTT-C14 film is shown in Figure 9. It is mostly dominated by C–H modes due to their large vibrational dipole moment and stretching vibrations from bonds in the thiophene ring [21,23,63,64,65,66]. The stretching vibration of bonds in the thiophene ring [23,66] and the stretching vibration of C–C bonds [66] produce peaks at 795 cm${}^{-1}$ and 1342 cm${}^{-1}$, respectively. The band between 1400 and 1600 cm${}^{-1}$ is related to the antisymmetric stretching vibration of C=C from the rings [65], the stretching vibration of C–C in aromatics [21], and the deformation vibration of –CH bonds [63,67]. The band between 1000 and 1140 cm${}^{-1}$ is related to the deformation vibration of C–H bonds [64], whereas the band between 3050 and 3150 cm${}^{-1}$ is due to the stretching vibration of C–H in aromatics [21]. Similarly, peaks at 2847 and 2916 cm${}^{-1}$ are a consequence of the stretching vibration of CH${}_{2}$ and CH${}_{3}$ bonds in alkanes [21,66]. Despite its investigation for poly-3-hexylthiophene (P3HT) by DeLongchamp et al. [64], a noticeable peak at 2916 cm${}^{-1}$ is also considered a sign of a well-organized semiconducting film. A summary of this discussion, including additional peaks, is provided as Supplementary Materials (see Table S2).

**Figure 10:**
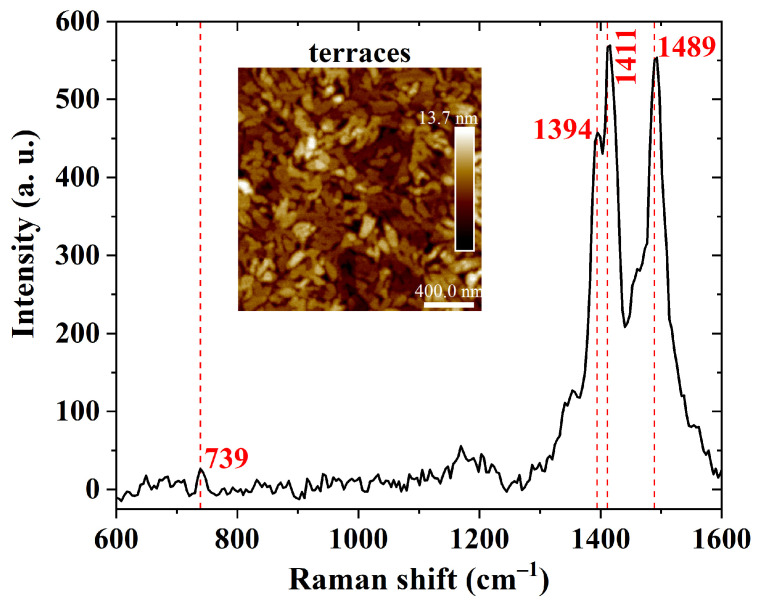
Raman shift of a 40 nm thick PBTTT-C14 film from a 6 mg/mL solution in DCB spun at 600 rpm/60 s over glass and treated according to H150N. Inset: AFM micrograph of the investigated semiconducting film.

A further investigation of carbon bonds in PBTTT-C14 films was performed using Raman spectroscopy, as shown in Figure 10. The peaks at 1489 cm${}^{-1}$ and 1394 cm${}^{-1}$ are due to stretching vibrations of C=C and C–C bonds in the thiophene ring, respectively [68,69,70]. The peak at 1411 cm${}^{-1}$ is due to stretching vibrations of C=C bonds in the thienothiophene ring [68,69,70]. Compared with previous results, differences in the shape of the curve and peak intensity are probably a consequence of differences in processing parameters. Therefore, solution heating and thermal annealing of deposited films did not chemically degrade the semiconducting polymer.

**Figure 11:**
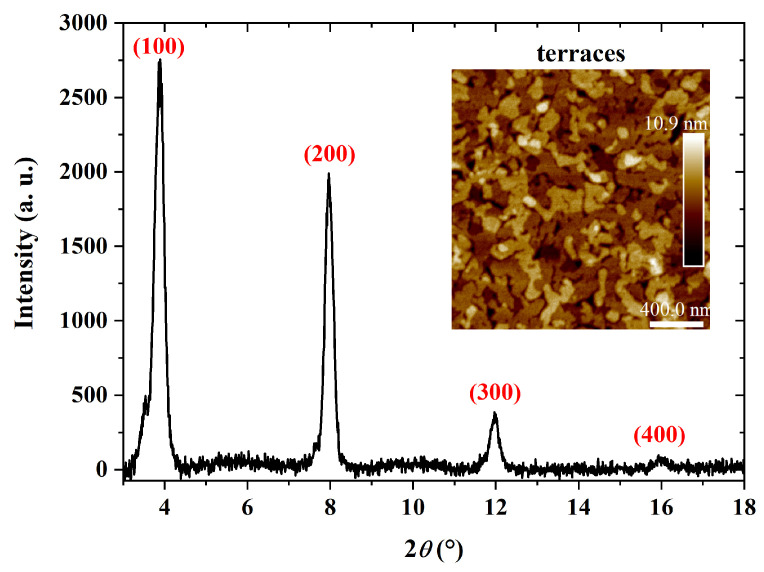
XRD of a 41 nm thick PBTTT-C14 film spun from a 6 mg/mL solution in DCB at 600 rpm/60 s over glass and treated according to E150. Inset: AFM micrograph of the investigated semiconducting film.

XRD results of PBTTT-C14 films with terraces on the surface are given in Figure 11. A *d* spacing of 22.2 Å was calculated from the peak at 2$*\theta =4^{\circ }$. According to Pandey et al. [68], a spacing of approx. 21 Å is expected along the stacking direction of alkyl groups. This value also represents the lamellar spacing, i.e., the distance neighboring conjugated backbones, positioned one on top of the other [54]. It can vary depending on the thermal treatment since side chains rearrange during recrystallization [68]. In addition, these results point to edge-on monomer positioning, i.e., perpendicular to the substrate [71]. In summary, XRD results also indicate that treatment both at 150 ${}^{\circ }$C and in a controlled atmosphere (e.g., low vacuum or under inert gas) enhances film crystallinity through the formation of terraces.

### 3.2. Validation of PVP:PMF Cross-Linking from Optical and Electrical Measurements

**Figure 12:**
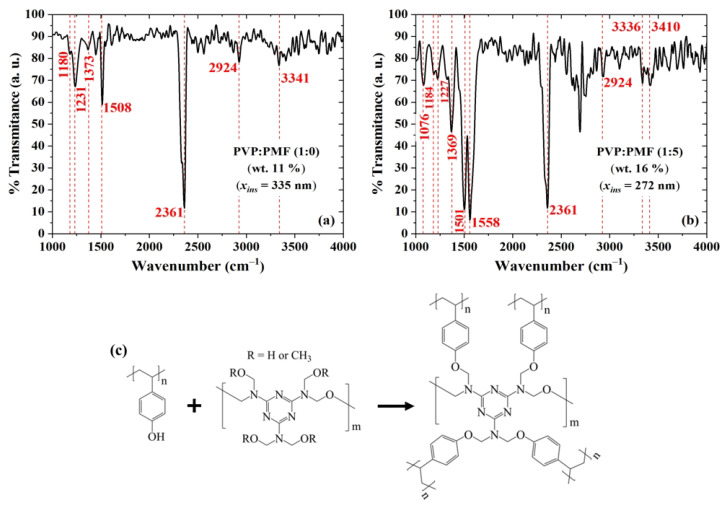
FTIR transmittance of PVP:PMF films spun at 2000 rpm/60 s from a PGMEA solution at (**a**) 1:0 and (**b**) 1:5 mass ratio over Si. (**c**) Illustration of the chemical reaction between PVP and PMF.

PVP and PMF polymers can undergo cross-linking when exposed to heat or catalysts. This process involves the reaction between the hydroxyl groups of PVP and the amino groups of PMF, resulting in the creation of cross-links [72,73,74]. Thermal cross-linking can occur through such reactions. This method induces chemical changes in the polymers when subjected to elevated temperatures, leading to the formation of a three-dimensional network structure. FTIR measurements in Figure 12 were taken in order to demonstrate the presence of cross-linking in PVP:PMF films. The results for the pure PVP films in Figure 12a show bands at 2924 cm${}^{-1}$ and 3200–3550 cm${}^{-1}$, which are assigned to the stretching vibration of C–H and O–H groups, respectively [75,76,77]. After the addition of PMF at a 1:5 mass ratio, as shown in Figure 12b, these bands become narrowed and tend to disappear. An exception is a band between 3336 cm${}^{-1}$ and 3410 cm${}^{-1}$, due to OR (R = H or CH${}_{3}$) groups from PMF [77] and hydrogen bonding between PVP and PMF of the remaining hydroxyl groups [78]. Other peaks arise at 1501 and 1558 cm${}^{-1}$ due to the bending vibrations of C–O bonds [75] and C–N from triazine rings [79], respectively. There is also a new peak at 1076 cm${}^{-1}$, which is related to the out-of-phase stretching modes of C–O–C bonds [79,80]. The presence of PMF in the film is also evidenced by an increase in the peak at 1369 cm${}^{-1}$, which is related to the presence of C–N groups [81]. All these changes after the addition of PMF point to enhanced cross-linking in the film. The peak at 2361 cm${}^{-1}$ is likely due to the presence of CO${}_{2}$ in the air during data acquisition [82]. Based on these results, the cross-linking reaction between PVP and PMF is illustrated in Figure 12c. It happens through PMF bonding to the phenol group in PVP, which decreases the number of hydroxyl groups in the film [75,76,77].

**Figure 13:**
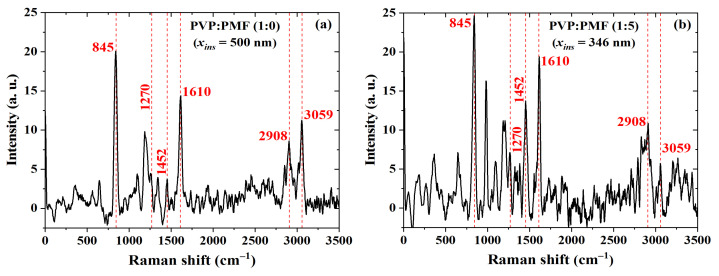
Raman shift of PVP:PMF films spun at 1000 rpm/60 s from a 16 wt.% PGMEA solution at (**a**) 1:0 and (**b**) 1:5 mass ratio over glass.

In order to acquire further evidence of a cross-linking reaction, these dielectric films were investigated using Raman spectroscopy. Typical PVP bands, as shown in Figure 13a, are present at 845 and 1610 cm${}^{-1}$ due to out-of-plane deformation of the C–H bonds and C–C bond vibration from the phenyl group, respectively [83]. The peak at 2908 cm${}^{-1}$, due to the tertiary C-H stretching vibration [84], increased after PMF addition, as shown in Figure 13b, whereas the band due to the aromatic C–H stretching vibration at 3059 cm${}^{-1}$ [84] almost disappeared. On the contrary, there was an increase in the bands at 1270 and 1452 cm${}^{-1}$ related to C–O–C [84,85] and C–N [79,85] groups, respectively. These are more indications of enhanced cross-linking in the dielectric film.

**Figure 14:**
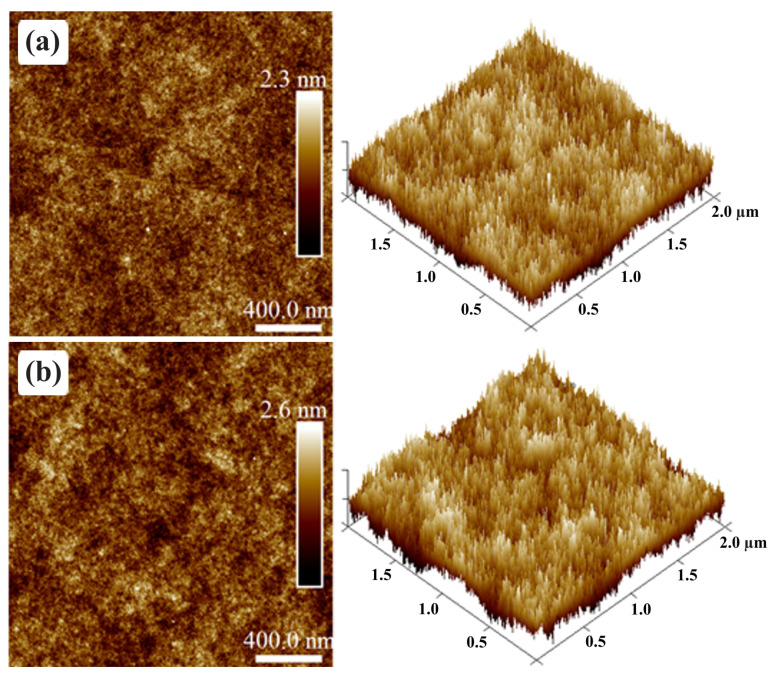
AFM micrographs of PVP:PMF film surface from a (**a**) 1:0 mass ratio and 6.3 wt.% in PGMEA and a (**b**) 1:5 mass ratio and 16 wt.% in PGMEA spun at 1000 rpm/60 s over glass.

Dielectric films were also investigated via AFM in order to ensure that their thickness and roughness were adequate for OTFT applications. The AFM of PVP-based films is shown in Figure 14. A 381 nm thick PVP film features an Rq of 0.3 nm in Figure 14a. On the other hand, after PMF addition at a 1:5 ratio, Rq slightly increases to 0.38 nm in Figure 14b. Despite an increase of 60% in the solution concentration in PGMEA for the same PVP mass, the film thickness increased just by ca. 17%, i.e., to 459 nm. This is an indirect sign of cross-linking, as such films tend to be more compact [74,86]. Ultimately, these thickness values are acceptable for OTFT fabrication (590–1150 nm) [10,87]. Dielectric surface roughness is even lower than previous reports on thinner PVP:PMF films [28]. According to Jung et al. [60], an Rq lower than 0.5 nm is desirable for large terrace formation in PBTTT-C14 films and, consequently, improved charge transport along the semiconductor.

**Figure 15:**
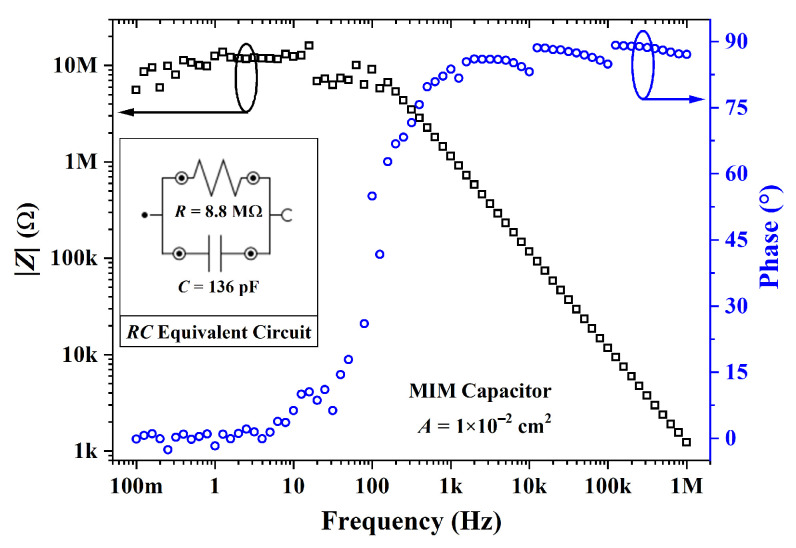
Impedance modulus and phase for an MIM capacitor from PVP:PMF ($x_{ins}=346$ nm) at a 1:5 ratio spun from a 16 wt.% solution in PGMEA over glass.

**Figure 16:**
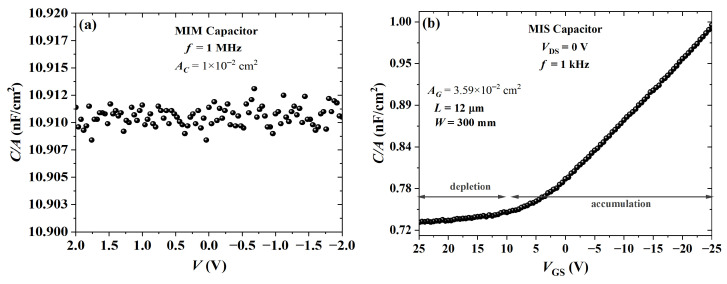
Capacitance versus voltage for PVP:PMF ($x_{ins}=413$ nm) at a 1:5 ratio spun at 2000 rpm from a 19 wt.% solution in PGMEA over glass: (**a**) MIM and (**b**) MIS with PBTTT-C14 ($x_{s}=15$ nm) spun at 1800 rpm from a 6 mg/mL solution in DCB and annealed according to H150N.

An MIM capacitor was processed from cross-linked PVP. The Bode diagram in Figure 15 shows that the impedance was ca. 10 MΩ and $0^{\circ }$ for frequencies in the range of 0.1 to 10 Hz. This purely resistive behavior gradually shifted to a purely capacitive one from 1 kHz to 1 MHz with an impedance phase of ca. $90^{\circ }$. By using a parallel RC circuit model, a dielectric constant of 5.29±0.15 was calculated. As expected, the film is a high *k*, which is in agreement with previous results [73,88,89]. The C-V curves of capacitors from cross-linked PVP are given in Figure 16. As expected, the capacitance from an MIM structure, as shown in Figure 16a, remained constant. In contrast, the addition of PBTTT-C14 in an MIS structure led to a lower capacitance with an increase in $V_{GS}$ (see Figure 16b). There was a noticeable change in capacitance with gate bias due to the additional capacitive element in series originating from the depleted semiconductor. This behavior could not be observed at high frequencies (e.g., 1 MHz) due to the low charge-carrier mobility from the semiconductor.

**Figure 17:**
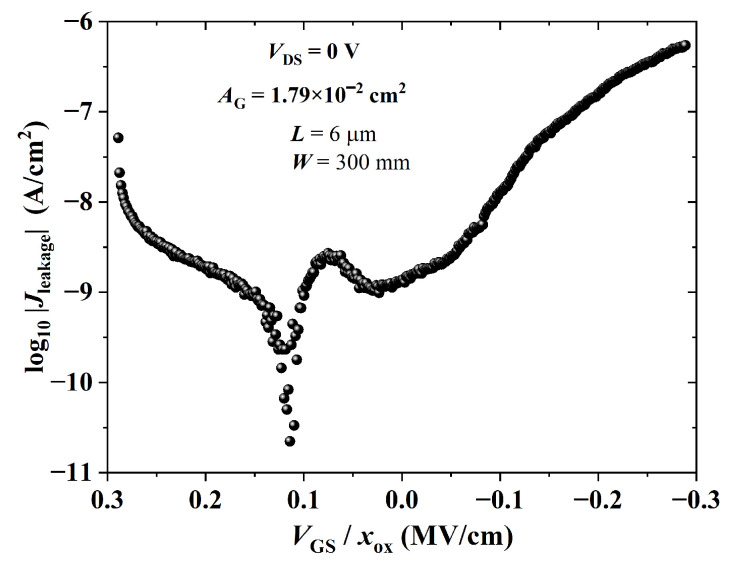
Leakage current density versus gate dielectric electric field of a BGBC-OTFT from PBTTT-C14 ($x_{s}=25$ nm) spun at 600 rpm from a 6 mg/mL solution in DCB and annealed according to H150N on top of PVP:PMF ($x_{ins}=346$ nm) at a 1:5 ratio spun at 2000 rpm from a 16 wt.% solution in PGMEA over glass.

The leakage current density through the gate dielectric as a function of the electric field is given in Figure 17. Its maximum absolute value was ca. 540 nA/cm${}^{2}$ at −0.3 MV/cm (i.e., an $I_{G}$ of approx. 9.8 nA at $V_{GS}=-10$ V and $V_{DS}=0$ V), which is critical for OTFT operation in the cut-off region. High off currents are common in high-*k* dielectrics, so a thin buffer layer is usually required for proper TFT operation [73,89]. Nevertheless, in this work, transistors were gas sensors operating in the triode region, in which the current in the channel (∼20–100 nA) was at least three orders of magnitude higher than the one through the gate dielectric (<20 pA).

### 3.3. PBTTT-C14 Transistor Characterization

**Figure 18:**
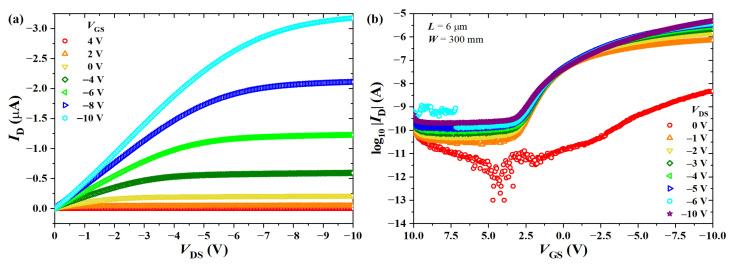
(**a**) Output and (**b**) transfer characteristics of a BGBC-OTFT from PBTTT-C14 ($x_{s}=25$ nm) spun at 600 rpm from a 6 mg/mL solution in DCB and annealed according to H150N on top of PVP:PMF ($x_{ins}=346$ nm) at a 1:5 ratio spun at 2000 rpm from a 16 wt.% solution in PGMEA over glass and in a N${}_{2}$-filled glovebox.

The output and transfer characteristic curves of OTFTs are given in Figure 18. Table 5 summarizes the electrical parameters extracted from the transistors under investigation. Devices were initially studied inside the glovebox in order to quantify the effect of exposure to oxygen and moisture. Pristine average values obtained in a N${}_{2}$ atmosphere were $\mu_{p,tri}=1.21\times 10^{-4}$ cm${}^{2}$/Vs, $\mu_{p,sat}=1.78\times 10^{-4}$ cm${}^{2}$/Vs, $V_{T}=0.80$ V, $I_{ON/OFF}=29,600$, and SS=−0.80 V/dec. Removal from the glovebox led to doping since mobility in the triode region increased by ∼700%, the threshold voltage shifted to positive values by ∼16.5%, current modulation decreased by ∼81%, and the subthreshold swing increased by ∼670%. This is a behavior usually observed in polythiophene derivatives [6,36,61,63,69]. It is worth noticing that water molecules also interact with the dielectric, causing a degradation in performance through $V_{T}$ and SS [74,89].

Doping and chemical degradation of organic layers upon exposure to the atmosphere outside of the glovebox was reduced by performing gas-sensing measurements in the dark, under reduced voltage stress, and by flowing pure nitrogen at low relative humidity (RH=15%). A careful bias stress investigation was carried out to assess the effect of electrical polarization on OTFT performance over time. These results can be found in detail in [37]. In short, there was a noticeable shift in the threshold voltage at high bias voltage (∼5 V). That is the reason why gas sensors were interrogated at −1 V $<V_{GS}<1$ V and $V_{DS}=-0.5$ V. In this way, it is assumed that variations in electrical parameters due to polarization stress, such as in mobility (μ), $V_{T}$, or channel current ($I_{ON}$), are negligible during the experiments.

### 3.4. Gas-Sensing Response

**Figure 19:**
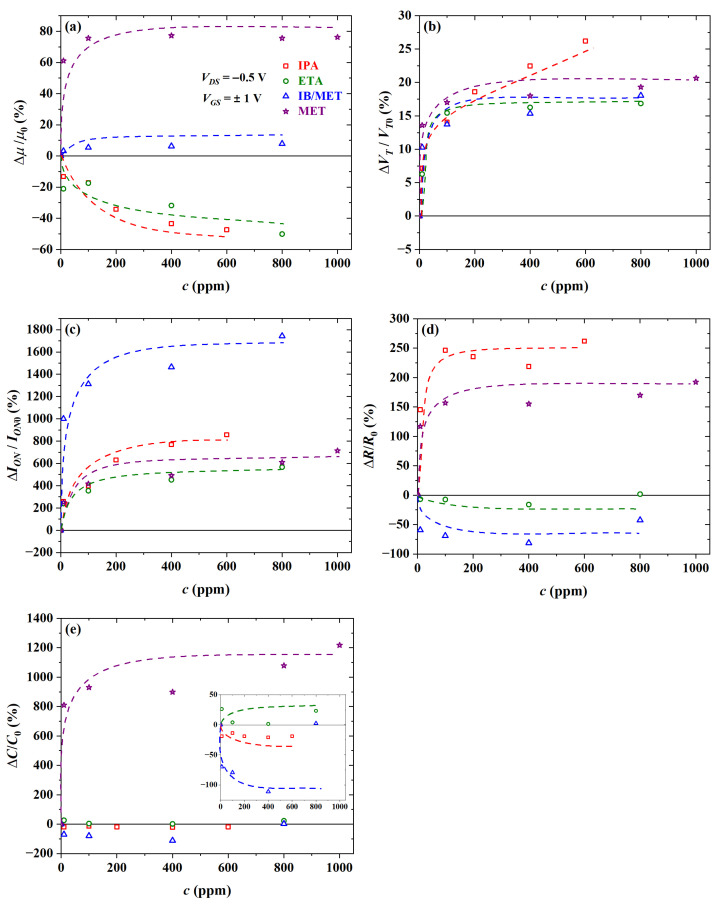
Relative variation in (**a**) μ, (**b**) $V_{T}$, and (**c**) $I_{ON}$ of transistors, as well as (**d**) *R* and (**e**) *C* of chemical sensors, as a function of analyte concentration in ppm.

The electrical parameters’ relative variation extracted from OTFTs’ transfer curves (see Figure S6 in the Supplementary Materials) is reported in Figure 19. Significant shifts for μ, $V_{T}$, and $I_{ON}$ at 10 ppm were observed in the presence of MET (∼61%), MET (∼14%), and IB/MET (∼1000%), respectively. Mobility increased in the presence of MET and IB/MET but decreased when exposed to IPA and ETA. Threshold voltage and channel current always increased in the presence of alcohols and isoborneol.

At low analyte concentrations, the transistors were highly responsive to methanol and isoborneol. Under similar ppm levels, *R* varied ca. 150% upon exposure to IPA, increasing both in the presence of IPA and MET, whereas decreasing in the presence of ETA and IB/MET. The capacitance, on the other hand, increased ca. 810% upon exposure to MET, increasing both in the presence of MET and ETA, whereas it decreased in the presence of IPA and IB/MET.

At high concentrations (i.e., approaching 1000 ppm), significant shifts for μ, $V_{T}$, and $I_{ON}$ were observed in the presence of MET (∼76%), IPA (∼26%), and IB/MET (∼1740%), respectively. There was a clear saturation profile for OTFT parameters above 100 ppm. In any case, the transistors were also highly responsive to isopropyl alcohol. At these concentration levels, *R* increased ca. ∼260% upon exposure to IPA, whereas *C* increased ca. 1220% in response to MET. Chemical sensors also tend to saturate for concentrations greater than 100 ppm.

Table 6 was generated in order to assess the effect of measuring isoborneol in the presence of methanol. Except for $V_{T}$ (∼3%), there was a noticeable difference between the response to IB/MET and pure MET. Chemical sensors can also clearly distinguish between these two analytes. As will be seen later, these conclusions are also supported by PCA and sensitivity data.

**Figure 20:**
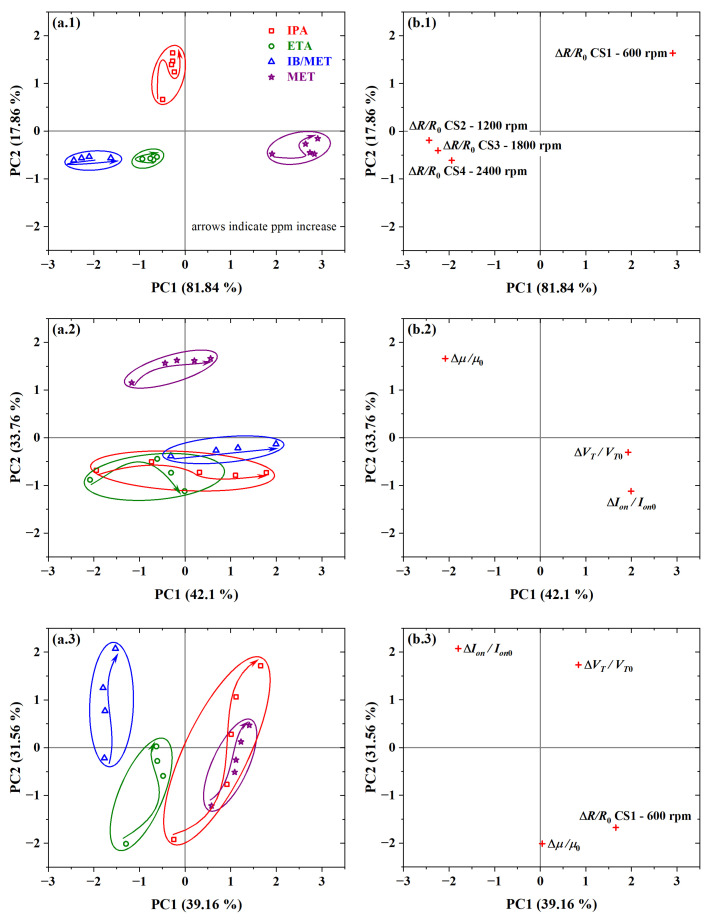
PCA plots from (**a.1**,**b.1**) resistance data of PBTTT-C14 interdigitated chemical sensors; (**a.2,b.2**) mobility, threshold voltage, and on current of PBTTT-C14 OTFTs; and (**a.3**,**b.3**) resistance, as well as mobility, threshold voltage and on current, together, in response to all analytes: (**a.1**–**a.3**) scores and (**b.1**–**b.3**) loadings data.

PCA plots are given in Figure 20. By using only chemical sensors’ resistance data, a PC1 of 81.84% and a sum (i.e., PC1+PC2) of 99.69% was observed (see scores in Figure 20(a.1)). It was possible to discriminate the four analyte samples into distinct clusters. In this case, there was also significant discrimination between the MET and IB/MET samples. This was the highest discrimination level observed for PCA bidimensional plots at hundreds of ppm of the investigated analytes.

The resistance of the chemical sensor from PBTTT-C14 spun at 600 rpm was detached from the ones from the other sensors (see loadings in Figure 20(b.1)). The analysis of the loadings plot indicates that CS1 contributed the most to the discrimination of IPA along the PC2 axis. Additionally, CS1 contributed inversely to CS2, CS3, and CS4 in the discrimination along the PC1 axis. This points to a significant contribution coming from CS1, which is probably due to the thicker film and slower drying originating from the lower spinning frequency during deposition. Compared with MET and ETA, IPA has a lower dielectric constant (20.18) and dipole moment (1.56 D) [91]. It is likely that IPA penetrates deeper into the semiconducting film thanks to voids in the grain boundaries in a more crystalline structure, such as from CS1. In addition, a rougher surface from thicker films such as CS1 provides more interaction sites toward the detection of gaseous analytes. Similar behavior would be expected for IB, since it has an even lower dielectric constant (3.3 [92]) and dipole moment (1.2 D [93]). In this case, however, the larger molecular size of IB (0.50 nm [94]) compared with the other analytes [95,96] might play a role against analyte diffusion into the sensing film.

The use or addition of capacitance data for the analysis worsens the discrimination ability of the system (see Figure S7 in the Supplementary Materials). This agrees well with the fact that a zoom-in on Figure 19e was necessary in order to distinguish among the response to IPA, ETA, and IB/MET. The higher selectivity of capacitance data to MET is probably related to its higher dielectric constant (33.0) and dipole moment (1.70 D) with respect to the other investigated analytes [91]. The average physicochemical properties of investigated analytes are summarized in Table S5 in the Supplementary Materials.

Performing a PCA with only the transistors’ electrical parameters, such as μ, $V_{T}$, and $I_{ON}$, provided a PC1 of 42.10% and a sum of 75.86% (see scores in Figure 20(a.2)). In this case, there was the most significant overlap of clusters from different analyte samples along the PC1, such that the use of two PC axes provided only partial discrimination. Despite that, it was possible to separate MET and IB/MET on opposite sides of the plot. In addition, there should be enough MET in the IB solution to saturate the sensor response to that analyte. In this work, for each 10 ppm of IB, there are 56 ppm of MET only (i.e., approx. five times more). Therefore, at 100 ppm of IB, there is already 560 ppm of MET, i.e., more than enough for the response to be saturated from methanol. This is another clear indication that PBTTT-C14 TFTs respond to isoborneol. The reduced discrimination compared with CS points to better use of transistors at lower concentration levels (i.e., from units to tens of ppm). However, it should be noted that TFTs provide at least three significant parameters: μ, $V_{T}$, and $I_{ON}$. At the concentration levels used in this work, there is no reasonable gain by combining CS and OTFT data. In this case, both the PC1 and the sum decreased to 39.16% and 70.72%, respectively (see scores in Figure 20(a.3)). For more details, see Table S4 in the Supplementary Materials.

The reproducibility of chemical sensors from PBTTT-C14 films was also demonstrated in this work (see Table S4 in the Supplementary Materials). The difference in the sum of the first two principal components was only 3.4% in PCA obtained from *R* data, 3.1% from *C*, and 0.4% from both *R* and *C*. The best combination of electrical parameters was achieved with *R*, μ, and $I_{ON}$, which provided a PC1 of 68.24% and a sum of ∼97.8%. One direct conclusion is that, despite the excellent attributes already described for the cross-linked dielectric, it interferes negatively with the discrimination of isoborneol through the OTFT’s threshold voltage.

**Figure 21:**
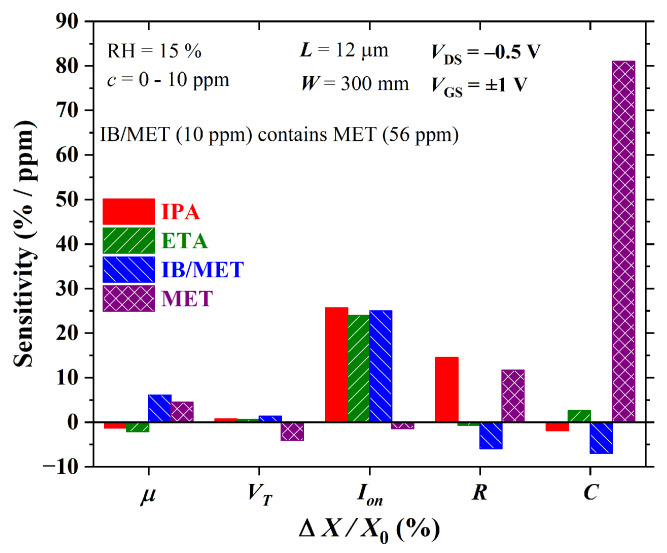
Sensitivity versus electrical parameter variation in both OTFT (μ, $V_{T}$, and $I{}_{O}N$) and chemical sensors (*R* and *C*) for analytes at 10 ppm concentration.

The sensitivity calculated at the 10 ppm level for both OTFT and CS is shown in Figure 21. Charge-carrier mobility enhances the discrimination of IB/MET and MET. It features a sensitivity of ca. 6.1% and 4.5%/ppm to IB/MET and MET, respectively. The threshold current and the channel current, on the other hand, assist mostly in the discrimination of MET with a sensitivity of ca. −4% and −1.4%/ppm, respectively. In agreement with an earlier discussion, $V_{T}$ is highly sensitive to methanol, which impairs the discrimination of isoborneol. As expected, *R* helps to separate IB/MET with a sensitivity of approx. 5.9%/ppm, despite being highly sensitive to IPA too (∼14.5%/ppm). *C* behaves similarly to $V_{T}$, being highly sensitive to MET (ca. 81%/ppm).

Multiple mechanisms of detection are believed to happen simultaneously within the organic material. For instance, polar molecules can become trapped in the PBTTT-C14 film and interact with charge carriers (i.e., holes) [57,97]. This interaction usually translates into a change in the potential barrier to transport and in the charge trapping along the channel. The analyte molecules are expected to physically adsorb onto the surface of the sensing semiconductor film, but also percolate through the voids surrounding the grains until they reach the dielectric surface [57]. Notably, the degree of physisorption depends on the chemical affinity between the active layer and the analyte, occurring both in the bulk of the semiconducting film and at the interface with the dielectric [57]. In summary, it has been shown that PBTTT-C14 is not only suitable for moisture and ammonia detection [23,37,98], but also has enormous potential for alcohol detection and water quality assessment.

## 4. Conclusions

The thermal treatment played a key role in the formation of terraces in PBTTT-C14 films. Among the possible microstructures, terraces were the most compact and, at the same time, had the largest grains. Only two drying procedures were able to generate terraces: (i) 150 °C for 20 min in a vacuum oven at 0.2 bar and (ii) 150 °C for 20 min on a hot plate inside a N${}_{2}$-filled glovebox with O${}_{2}$ and moisture levels under 10 ppm. The semiconducting films considered suitable for gas-sensor fabrication were spun at 600 rpm from 10 mg/mL solutions in dichlorobenzene. These films were 65 nm thick and 2 nm rough. The cross-linking of PVP:PMF (1:5 mass ratio) annealed at 175 °C for 120 min was confirmed via FTIR and Raman spectroscopies. These techniques also demonstrated that processing parameters such as solution heating and thermal treatment after deposition did not chemically degrade the polymer molecules. The cross-linking could be further demonstrated with capacitance measurements. In addition, a high dielectric constant of approx. five and a leakage current of ca. 20 pA at 1 V were adequate for low-voltage sensor operation.

The fabricated bottom-gate/bottom-contact OTFTs were sensitive to isoborneol. In the presence of analytes in the gas phase, the transistor current was the parameter that featured significant shifts with approx. 1740% in response to 800 ppm of isoborneol and 710% to 1000 ppm of methanol. The device has the potential to assist in the detection of other alcohol molecules, such as ethanol and isopropyl alcohol. Principal component analysis of data from PBTTT-C14-based sensors demonstrated that an e-nose composed solely of chemical sensors was able to discriminate all analytes in the hundreds of ppm. The addition of OTFT data to the PCA should be promising for discrimination at lower analyte concentrations (<10 ppm). The capacitance of chemical sensors was shown to be highly sensitive to methanol (ca. 81%/ppm). Finally, the resistance was shown to be adequate for the detection of all analytes in the hundreds of ppm. The results herein demonstrate a huge potential for this material to compose an e-nose for the detection of alcohol molecules, such as methylisoborneol, found in contaminated water.

## Figures and Tables

**Table 1:** List of chemical sensors from PBTTT-C14 in DCB spun on top of Ni:Cr/Au interdigitated electrodes over glass.

Device	*c* (mg/mL)	*f* (rpm)	Drying Procedure
CS1	10	60	H150N
CS2		1200	
CS3		1800	
CS4		2400	

**Table 2:** Fitting parameters for the thickness depending on solution concentration of PBTTT-C14 films spun over glass and dried overnight under ambient conditions.

*f* (rpm)	$K_{1}$ nm (mg/mL)${}^{-K_{2}}$	$K_{2}$ (a.u.)
600	1.90±0.15	1.60±0.05
1200	2.30±0.20	1.10±0.05
1800	1.80±0.20	1.10±0.07
2400	1.59±0.30	1.03±0.12
3000	0.86±0.16	1.38±0.11

**Table 3:** Fitting parameters for the thickness depending on spinning frequency of PBTTT-C14 films spun from 6 mg/mL solution in DCB over glass.

Annealing Process	$M_{1}$ nm (rpm)^−*M*_2_^	$M_{2}$ (a.u.)
A	5161±3361	−0.80±0.09
E80	2410±2476	−0.70±0.14
H150	2709±917	−0.70±0.05

**Table 4:** Summary of AFM-measured thickness and RMS roughness for different annealing processes of PBTTT-C14 films spun from a solution in DCB.

Annealing Process	*c* (mg/mL)	*T* (${}^{\circ }$C)	$x_{s}$ (nm)	Rq (nm)	Surface Structures
A	6	25	33±3	1.6±0.1	amorphous
EA			27±2	2.2±0.2	amorphous
H80		80	28±1	2.7±0.1	nodules
E80			29±3	2.0±0.2	amorphous
H150		150	30±2	2.4±0.1	nodules
E150			41±3	3.2±0.1	terraces
H150N			25±5	1.9±0.2	terraces
H150N	8	150	48±2	1.5±0.1	terraces

**Table 5:** Summary of electrical parameters extracted from transistors on glass, characterized both inside and outside the glovebox. Adapted from [90].

Dielectric	Semicond.	$\mu_{p,\mathrm{tri}}\times 10^{4}$	$\mu_{p,\mathrm{sat}}\times 10^{5}$	$V_{T}$	$I_{\mathrm{ON}/\mathrm{OFF}}$	SS
**$x_{\mathrm{ins}}$ (nm)**	**$x_{s}$ (nm)**	**(cm${}^{2}$/Vs)**	**(cm${}^{2}$/Vs)**	**(V)**	**(A/A)**	**(V/dec.)**
PVP:PMF (1:5) 346±17	PBTTT-C14 ^1^ 25±3	1.21±0.21	17.80±3.10	0.80±0.14	29,600±5100	−0.80±0.14
PVP:PMF (1:5) 830±14	PBTTT-C14 ^2^ 65±5	8.20±0.70	2.73±0.23	17.25±1.47	564±48	−6.15±0.52

^1^ Inside the glovebox. Channel length of 6 ± 1 µm. ^2^ Outside the glovebox. Channel length of 12 ± 1 µm.

**Table 6:** Estimated average shift in electrical parameter at 100 ppm pure isoborneol concentration.

Electrical Parameter	IB/MET (%)	MET (%)	Difference (%)
$\Delta \mu /\mu_{0}$	5	76	−71
$\Delta V_{T}/V_{T0}$	14	17	−3
$\Delta I_{ON}/I_{ON0}$	1310	420	890
$\Delta R/R_{0}$	−70	160	−230
$\Delta C/C_{0}$	−80	930	−1010

## Data Availability

Not applicable.

## Supplementary Materials

The following supporting information can be downloaded at: https://www.mdpi.com/article/10.3390/s23187981/s1, Figure S1: Photomasks for (a) gate and (b) source/drain electrodes. The interdigitated structure is partially magnified for better visualization (only 5 pairs of digits are shown); Figure S2: The photomask of electrodes for a pair of chemical sensors. The interdigitated structure is partially magnified for better visualization (only 5 pairs of digits are shown); Figure S3: Illustrated photograph of the gas measurement system; Figure S4: Resistance and capacitance measurement system for chemical sensors; Figure S5: Photographs of the OTFT characterization box: (a) open chamber with a sample containing OTFTs; (b) closed chamber featuring electrode selectors, gas inlet, and gas outlet connections; Figure S6: Transfer characteristics of a BGBC-OTFT over glass in response to (a) MET, (b) ETA, (c) IPA, and (d) IB for gas-sensing analysis. PBTTT-C14 ($x_{s}=65$ nm) was spun at 600 rpm from a 10 mg/mL solution in DCB and annealed according to H150N, whereas PVP:PMF ($x_{ins}=830$ nm) at a 1:5 ratio was spun at 2000 rpm from a 25 wt.% solution in PGMEA; Figure S7: PCA plot graphs of (a) scores and (b) loadings from: (1) capacitance; (2) resistance and capacitance from PBTTT-C14 interdigitated chemical gas sensors; Figure S8: PCA plot graphs of (a) scores and (b) loadings from data of PBTTT-C14 gas-sensing devices: (1) capacitance of chemical sensors, as well as mobility, threshold voltage, and on current of OTFTs; (2) resistance and capacitance of chemical sensors, as well as mobility, threshold volt-age, and on current of OTFTs; Figure S9: PCA plot graphs of (a) scores and (b) loadings from: (1) resistance; (2) capacitance; and (3) resistance and capacitance from PBTTT-C14 interdigitated chemical gas sensors; Table S1: Wavelengths at peak and shoulder calculated from the absorbance spectra of PBTTT-C14 films spun at 600 rpm/60 s from 6 mg/mL solution in DCB over glass for different annealing processes; Table S2: Peaks from FTIR spectra of PBTTT-C14 films; Table S3: List of replicated chemical sensors from PBTTT-C14 in DCB spun on top of Ni:Cr/Au interdigitated electrodes over glass; Table S4: Summary of calculated Principal Components (PCs) from all PCA analyses performed in this work; Table S5: Average physicochemical properties of investigated analytes.
