# Supplementary material for: Detection of Water Contaminants by Organic Transistors as Gas Sensors in a Bottom-Gate/Bottom-Contact Cross-Linked Structure
Source: Sensors (Basel). 2023 Sep 20;23(18):7981. doi: 10.3390/s23187981 (PMC10534344; doi:10.3390/s23187981)
Supplement: Supplementary file 1 [file sensors-23-07981-s001.zip › sensors-2555320-supplementary.pdf]

sensors

MDPI

# Supplementary Material

## 1. Semiconducting polymer solution

The concentration of poly(2,5-bis(3-tetradecyl-thiophen-2-yl)thieno[3,2-b]thiophene) (PBTTT-C14) semiconducting polymer in dichlorobenzene (DCB) varied from 2 to 10 mg/mL according to the following:

- 2, 4, and 6 mg/mL for thickness and roughness using atomic force microscopy (AFM);
- 6 and 8 mg/mL for topography via AFM;
- 6 mg/mL for absorbance via UV-visible spectrophotometry;
- 10 mg/mL for transmittance via Fourier transform infrared (FTIR) spectroscopy;
- 6 mg/mL for intensity via Raman spectroscopy;
- 6 mg/mL for intensity via X-ray diffraction (XRD);
- 6 mg/mL for capacitance and leakage current from Metal–Insulator–Semiconductor (MIS) capacitors;
- 6 and 10 mg/mL for transistor performance from organic thin-film transistors (OTFTs);
- 6 and 10 mg/mL for sensor performance from OTFTs and chemical sensors.

Initially, an extended concentration range spanning from 2 to 8 mg/mL was utilized for AFM measurements. This range facilitated the creation of terraces, acknowledged to be conducive to optimal transistor performance [1–3]. Based on the observation of terrace formation from films spun from 6 mg/mL solutions, this specific concentration was adopted for a range of analyses, encompassing UV-vis, Raman, XRD, capacitor, transistor, and sensor experiments. It is worth noting that a deviation from this concentration was initially observed during FTIR spectroscopy. This necessitated the utilization of thicker films, prepared from solutions with a concentration of 10 mg/mL. This adjustment was prompted by the observed interference of noise in the data under normal conditions. Finally, this higher concentration solution was also employed for the fabrication of OTFTs used as gas sensors. This deliberate choice was made with the intent of amplifying the device’s current when these sensors were subjected to external environmental conditions outside of the controlled glovebox environment, where the risk of degradation was a concern.

## 2. Electrode geometry for OTFTs and chemical sensors

The photomask of the gate and source/drain electrodes for OTFTs is given in Figure S1.

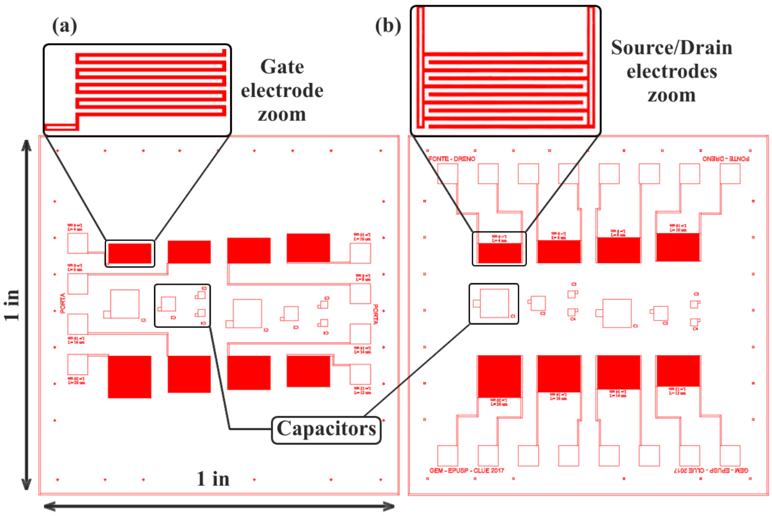


**Figure S1.** Photomasks for **(a)** gate and **(b)** source/drain electrodes. The interdigitated structure is partially magnified for better visualization (only 5 pairs of digits are shown).

The photomask of interdigitated electrodes for chemical sensors is given in Figure S2.

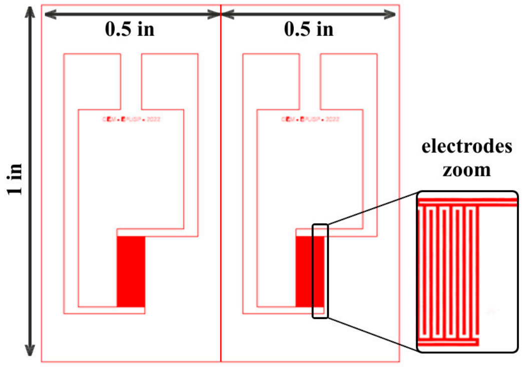


**Figure S2.** The photomask of electrodes for a pair of chemical sensors. The interdigitated structure is partially magnified for better visualization (only 5 pairs of digits are shown).

## 3. Gas measurement setup for OTFTs and chemical sensors

The apparatus was constructed within a fume hood to ensure human safety. It consisted of a bubbler to generate a moist $N_2$ flow and a gas mixer to manipulate the relative humidity level. Flow rates were regulated by means of rotameters, pressure regulators, and valves. The generated flows were directed to distinct chambers: one designated for OTFTs and the other for chemical sensors. Temperature and humidity gauges were positioned at the outlet.

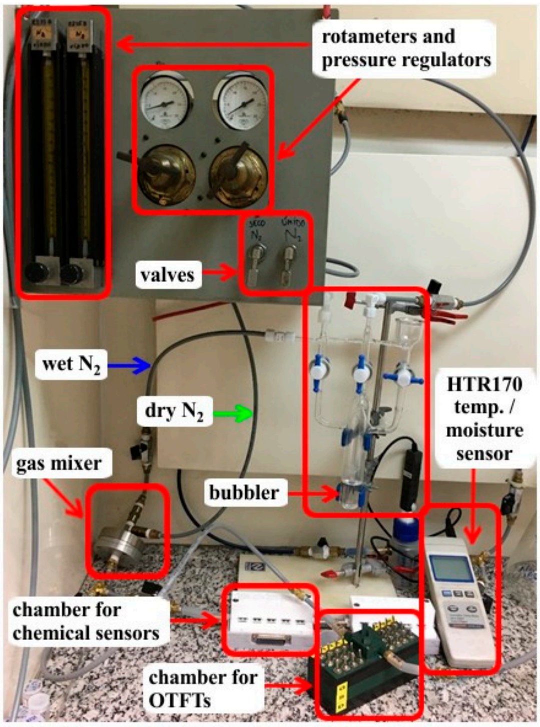


**Figure S3.** Illustrated photograph of the gas measurement system.

Chemical sensors were connected to a multiplexer (MUX) via a flat cable and a DB25 connector. The MUX was also connected to an LCR Meter Keysight and a computer for full measurement automation. The LCR Meter was connected to the computer, too, in which LabVIEW software was responsible for taking the measurements (see Figure S4).

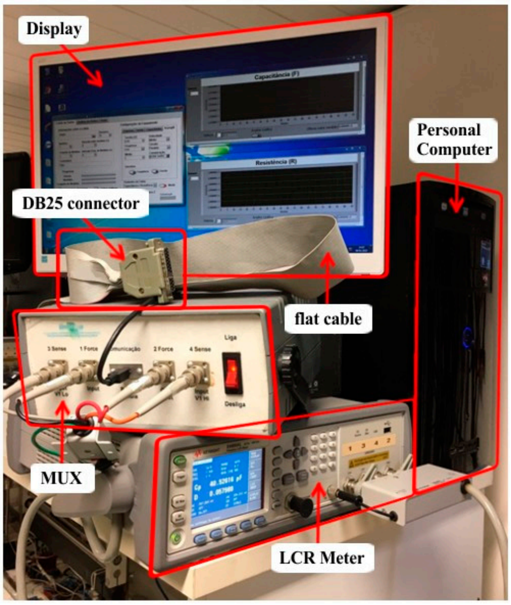


**Figure S4.** Resistance and capacitance measurement system for chemical sensors.

## 4. Gas measurement setup for OTFTs

The manual multiplexer and gas chamber for OTFTs, shown in Figure S5, were designed using AutoCAD software. They were fabricated in a 3D printer Da Vinci 1.0 Pro model of an XYZ Printing with PLA plastic.

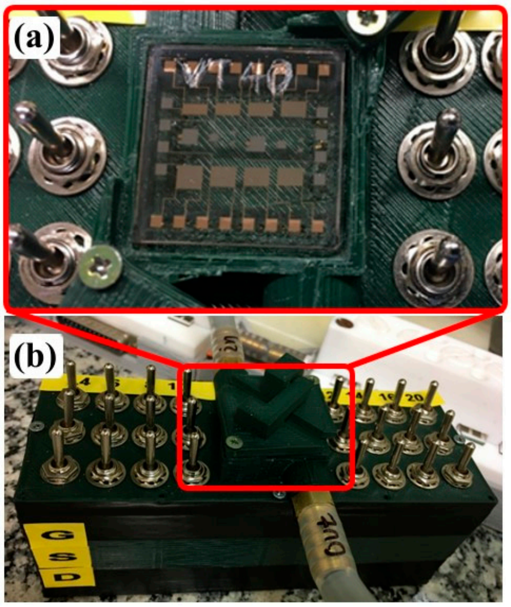


**Figure S5.** Photographs of the OTFT characterization box: **(a)** open chamber with a sample containing OTFTs; **(b)** closed chamber featuring electrode selectors, gas inlet, and gas outlet connections.

## 5. UV-visible data treatment

The obtained wavelengths at peak ($\lambda_{cp}$) and shoulder ($\lambda_{sh}$) values for PBTTT-C14 films annealed under different conditions are summarized in Table S1.

**Table S1.** Wavelengths at peak and shoulder calculated from the absorbance spectra of PBTTT-C14 films spun at 600 rpm/60 s from 6 mg/mL solution in DCB over glass for different annealing processes.

| Drying procedure | $\lambda_{cp}$ (nm) | $\lambda_{sh}$ (nm) |
|---|---|---|
| A | 551 | 585 |
| EA | 549 | 585 |
| E80 | 552 | 583 |
| E150 | 552 | 585 |
| H80 | 548 | 587 |
| H150 | 549 | 585 |
| H150N | 552 | 586 |
| A | 551 | 585 |

## 6. Peaks in the FTIR spectra from PBTTT-C14 films

A detailed list of the peaks extracted from the FTIR spectra of PBTTT-C14 films is provided in Table S2.

**Table S2.** Peaks from FTIR spectra of PBTTT-C14 films.

| Peak ($cm^{-1}$) | Description | Reference |
|---|---|---|
| 718–726 | rocking vibration of $CH_2$ alkyl | [4,5] |
| 780–795 | stretching vibration of the thiophene ring | [6,7] |
| 818–820 | wagging vibration of C = C–H along $\pi^*$ | [8] |
| | bending vibration of -CH out of the plane of the ring | [5] |
| 875 | out-of-plane deformation of C–H from the ring | [9] |
| 1,000–1,140 | deformation vibration of C–H | [8] |
| 1,180 | stretching vibration of C–C | [4] |
| 1,250–1,350 | stretching vibration of C–C | [6] |
| 1340 | wagging vibration of $–CH_2$ | [4] |
| | deformation vibration of –CH | |
| | symmetric deformation vibration, bending of $CH_3$ | |
| | twisting, torsion vibration of $CH_2$ | |
| 1,400–1,600 | stretching vibration of C–C in aromatics | [10] |
| 1,456 | deformation vibration of –CH | [5] |
| 1,466–1,470 | deformation vibration of $CH_2$ | [4] |
| | antisymmetric deformation vibration, bending of $CH_3$ | |
| 1,510 | stretching vibration of C–C in the backbone | [5] |
| 1,517 | antisymmetric stretching vibration of C=C from the rings | [9] |
| 1,550–1,650 | symmetric stretching vibration of C=C | [6] |
| | stretching vibration of C=C | [7] |
| 2,800–3,000 | stretching vibration of C–H from the thiophene ring | [6,7] |
| 2,850–3,000 | stretching vibration of C–H in alkanes | [10] |
| 2,847 | symmetric stretching vibration of $CH_3$ | [4] |
| 2,858–2,926 | stretching vibration of $CH_2$ alkyl | [5] |
| 2,916–2,919 | stretching vibration of cis and trans $CH_2$ | [4] |
| | asymmetric stretching of $CH_2$ | [7] |
| 3,050–3,150 | stretching vibration of C–H in aromatics | [10] |

## 7. Transfer characteristics of OTFTs in gas-sensing experiments

The drain current ($I_D$) versus gate-to-source voltage ($V_{GS}$) transfer curves of OTFTs in response to each analyte (MET, ETA, IPA, and IB) are elucidated in Figure S6. These plots have been employed for the explicit purpose of ascertaining the gas-sensing response. Specifically, they facilitate the extraction of individual device parameters ($\mu$, $V_T$, and $I_{ON}$) at each experimental concentration.

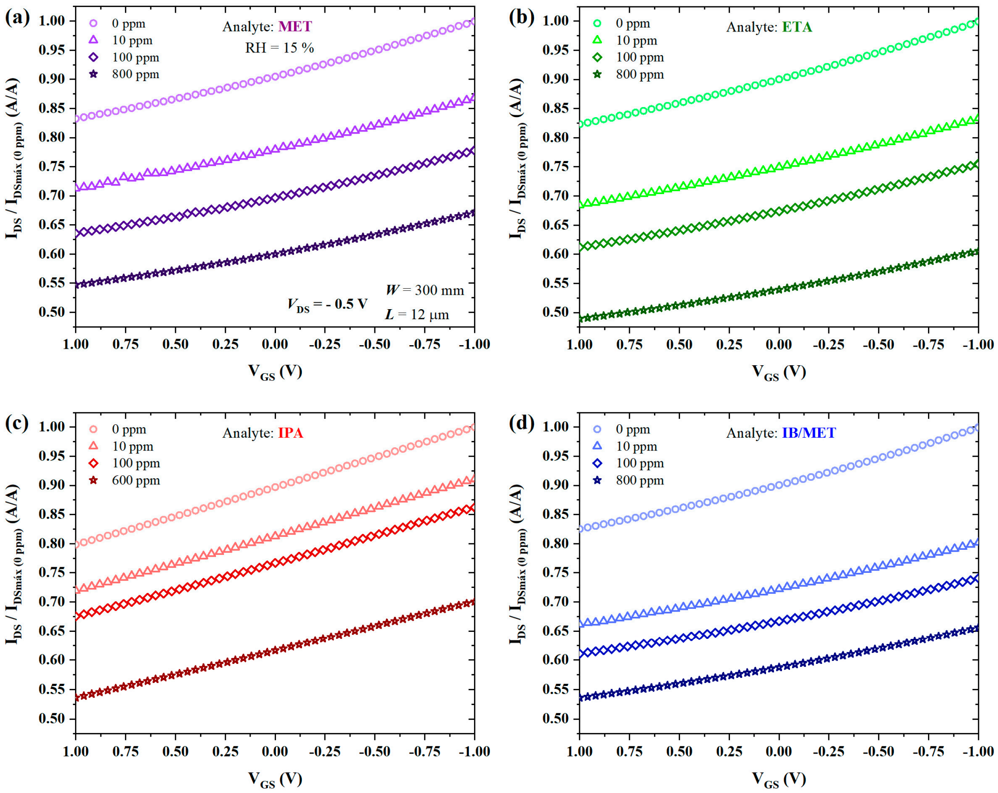


**Figure S6.** Transfer characteristics of a BGBC-OTFT over glass in response to **(a)** MET, **(b)** ETA, **(c)** IPA, and **(d)** IB for gas-sensing analysis. PBTTT-C14 ($x_s$ = 65 nm) was spun at 600 rpm from a 10 mg/mL solution in DCB and annealed according to H150N, whereas PVP:PMF ($x_{ins}$ = 830 nm) at a 1:5 ratio was spun at 2,000 rpm from a 25 wt.% solution in PGMEA.

## 8. PCA plots from gas-sensing results

PCA plots display scores from analyte samples and loadings from sensor electrical parameters. Data from chemical sensors are shown in Figure S7. The use of capacitance data impairs the discrimination of IB from IPA. After adding capacitance to resistance data, it became impossible to separate IPA from ETA instead.

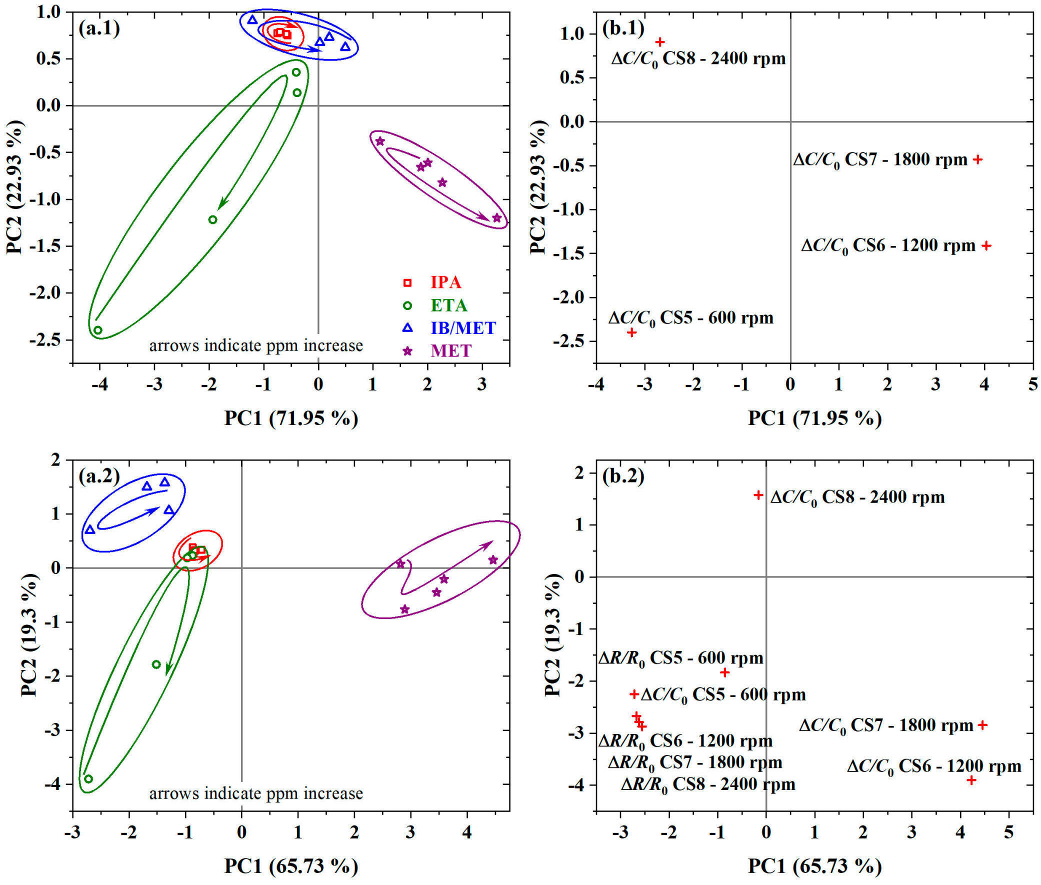


**Figure S7.** PCA plot graphs of **(a)** scores and **(b)** loadings from: **(1)** capacitance; **(2)** resistance and capacitance from PBTTT-C14 interdigitated chemical gas sensors.

PCA plots generated after the addition of OTFT data to the plots in Figure S7 are given in Figure S8. Discrimination is clearly improved, just in the case of having both resistance and capacitance data from chemical sensors, as well as mobility, threshold voltage, and on current from OTFTs. This was expected, since, in this case, all eligible electrical parameters from this work were used.

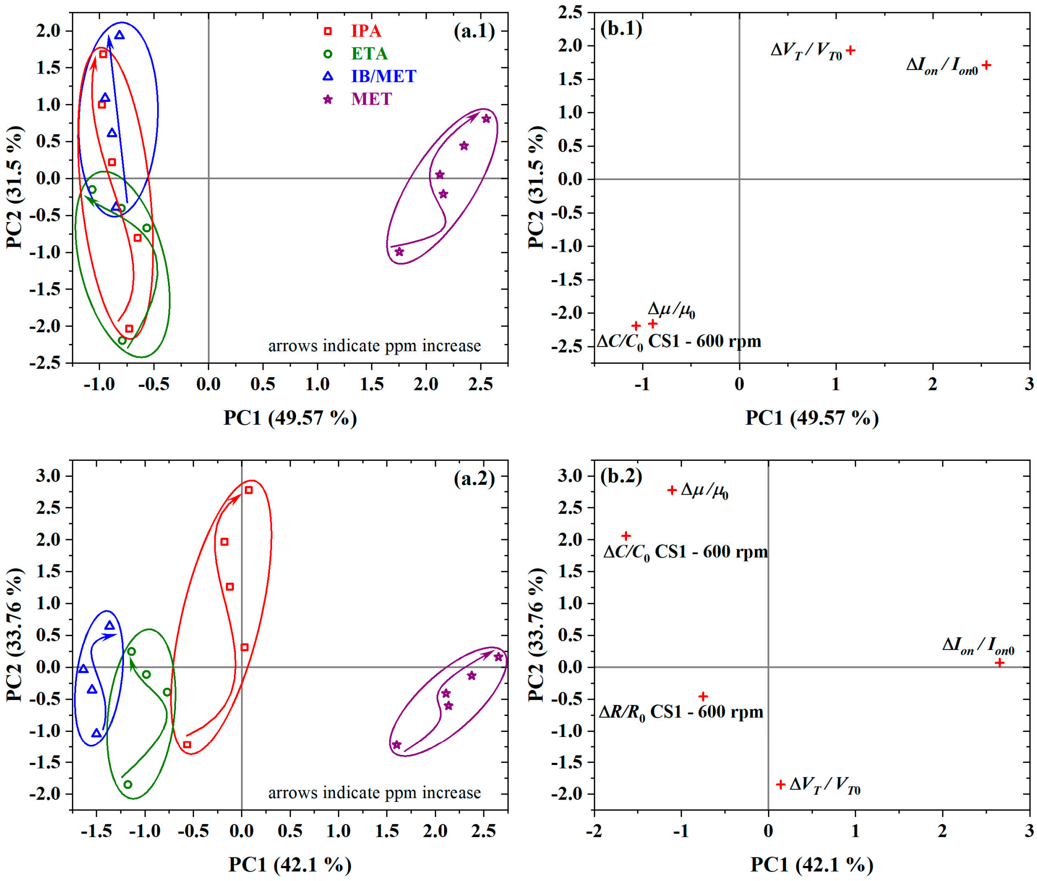


**Figure S8.** PCA plot graphs of (a) scores and (b) loadings from data of PBTTT-C14 gas-sensing devices: (1) capacitance of chemical sensors, as well as mobility, threshold voltage, and on current of OTFTs; (2) resistance and capacitance of chemical sensors, as well as mobility, threshold voltage, and on current of OTFTs.

In order to assess the reproducibility of the results with chemical sensors, replicas were fabricated according to Table S3.

**Table S3.** List of replicated chemical sensors from PBTTT-C14 in DCB spun on top of Ni:Cr/Au interdigitated electrodes over glass.

| Device | $c$ (mg/mL) | $f$ (rpm) | Drying procedure | Replicated from |
|---|---|---|---|---|
| CS5 | 600 | 585 | H150N | CS1 |
| CS6 | 1,200 | 585 | | CS2 |
| CS7 | 1,800 | 583 | | CS3 |
| CS8 | 2,400 | 585 | | CS4 |

The PCA plots obtained from the replicas of chemical sensors are given in Figure S9. Also, in this case, the best results were obtained by using just the resistance data. The addition of capacitance measurements did not enhance analyte discrimination, with a clear overlapping of scores from ETA and IPA. This agrees well with Figure S8. A summary of all PCA performed in this work is provided in Table S4.

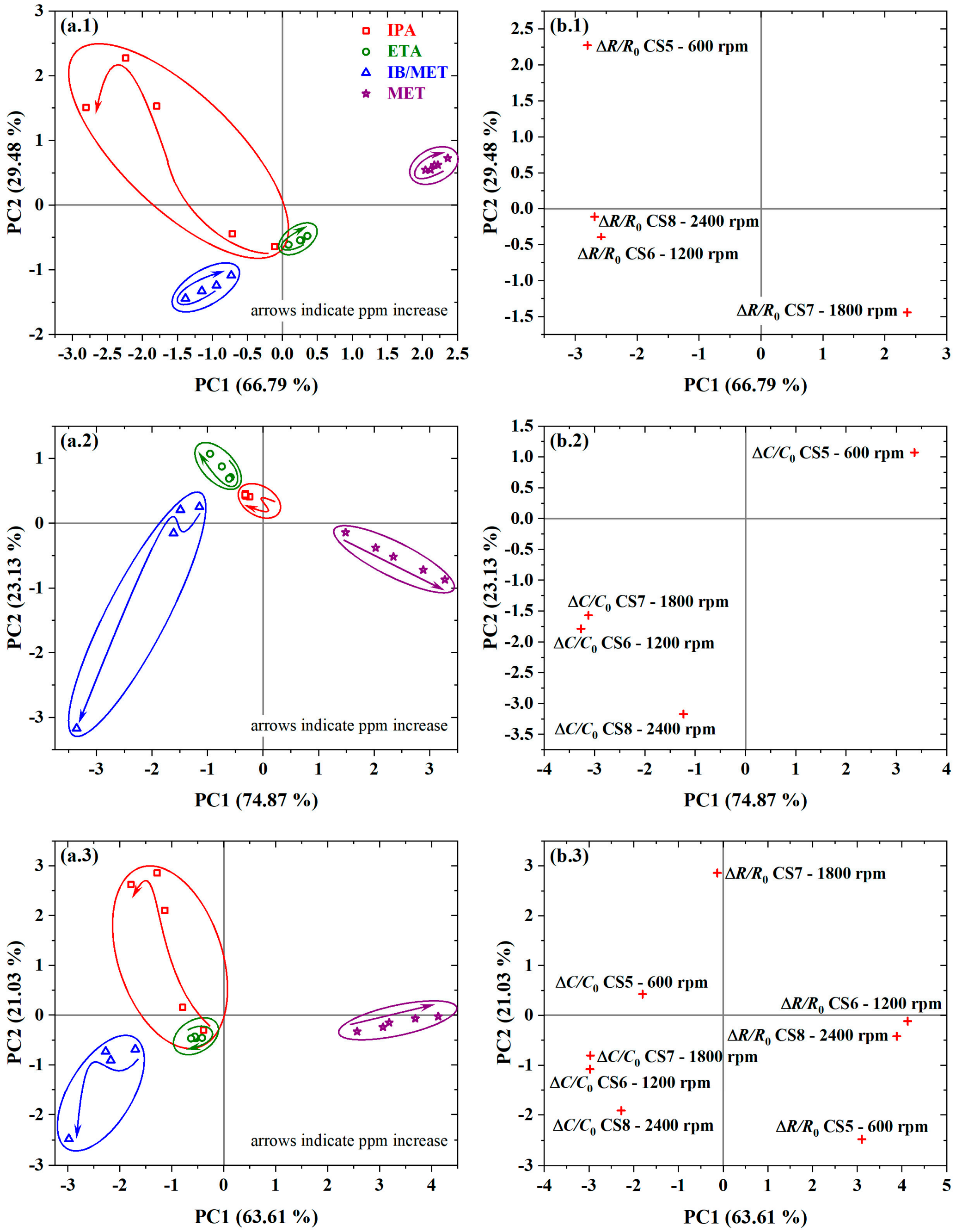


**Figure S9.** PCA plot graphs of (a) scores and (b) loadings from: (1) resistance; (2) capacitance; and (3) resistance and capacitance from PBTTT-C14 interdigitated chemical gas sensors.

**Table S4.** Summary of calculated Principal Components (PCs) from all PCA analyses performed in this work.

| Electrical parameter | Sensors | *PC1* (%) | *PC2* (%) | *Total* (%) |
|---|---|---|---|---|
| $R$ | CS1-CS4 | 81.84 | 17.86 | 99.69 |
| $C$ | CS1-CS4 | 71.95 | 22.93 | 94.88 |
| $R$; $C$ | CS1-CS4 | 65.73 | 19.30 | 85.03 |
| $\mu$; $V_T$; $I_{ON}$ | OTFT1 | 42.10 | 33.76 | 75.87 |

| | | | | |
|---|---|---|---|---|
| *R*; $\mu$; $V_T$; $I_{ON}$ | CS1; OTFT1 | 39.16 | 31.56 | 70.72 |
| *C*; $\mu$; $V_T$; $I_{ON}$ | CS1; OTFT1 | 49.57 | 31.50 | 81.07 |
| *R*; *C*; $\mu$; $V_T$; $I_{ON}$ | CS1; OTFT1 | 44.81 | 26.00 | 70.81 |
| R | CS5-CS8 | 66.79 | 29.48 | 96.27 |
| C | CS5-CS8 | 74.87 | 23.13 | 98.00 |
| R; C | CS5-CS8 | 63.61 | 21.03 | 84.64 |

The average values of static electric dipole polarizabilities, molecular sizes, dielectric constants, and dipole moments of investigated analytes are summarized in Table S5.

**Table S5.** Average physicochemical properties of investigated analytes.

| Analyte | Polarizability ($\times 10^{-24}$ cm$^3$) | Molecular size (nm) | Dielectric constant at 20 °C | Dipole moment (D) |
|---|---|---|---|---|
| Water | 1.45 [11] | 0.27 [12] | 80.10 [11] | 1.85 [11] |
| Methanol | 3.29 [11] | 0.43 [13] | 33.0 [11] | 1.70 [11] |
| Ethanol | 5.41 [11] | 0.44 [13] | 25.3 [11] | 1.69 [11] |
| Isopropyl alcohol | 7.61 [11] | 0.47 [13] | 20.18 [11] | 1.56 [11] |
| Isoborneol | 18.2 [14] | 0.50 [15] | 3.3 [16] | 1.2 [14] |

## References


1. Xue, X.; Chandler, G.; Zhang, X.; Kline, R.J.; Fei, Z.; Heeney, M.; Diemer, P.J.; Jurchescu, O.D.; O'Connor, B.T. Oriented Liquid Crystalline Polymer Semiconductor Films with Large Ordered Domains. *ACS Appl. Mater. Interfaces* **2015**, *7*, 26726–26734, doi:10.1021/acsami.5b08710.
2. DeLongchamp, D.M.; Kline, R.J.; Jung, Y.; Germack, D.S.; Lin, E.K.; Moad, A.J.; Richter, L.J.; Toney, M.F.; Heeney, M.; McCulloch, I. Controlling the Orientation of Terraced Nanoscale "Ribbons" of a Poly(thiophene) Semiconductor. *ACS Nano* **2009**, *3*, 780–787, doi:10.1021/nn800574f.
3. Wang S.; Tang, J.-C.; Zhao, L.-H.; Png, R.-Q.; Wong, L.-Y.; Chia, P.-J.; Chan, H.S.O.; Ho, P.K.-H.; Chua, L.-L. Solvent effects and multiple aggregate states in high-mobility organic field-effect transistors based on poly(bithiophene-alt-thienothiophene). *Appl. Phys. Lett.* **2008**, *93*, 162103, doi:10.1063/1.3001574.
4. Arndt, K.-F.; Lechner, M.D. *Landolt-Börnstein: Numerical Data and Functional Relationships in Science and Technology - New Series. Subvolume A: Polymer Solids and Polymer Melts. Part 1: Definitions and Physical Properties I. Group VIII: Condensed Matter Volume 6, 1st ed.*; Springer, **2013**; pp. 154–196.
5. Zhuo, J.-M.; Zhao, L.-H.; Png, R.-Q.; Wong, L.-Y.; Chia, P.-J.; Tang, J.-C.; Sivaramakrishnan, S.; Zhou, M.; Ou, E.C.-W.; Chua, S.-J. et al. Direct Spectroscopic Evidence for a Photodoping Mechanism in Polythiophene and Poly(bithiophene-alt-thienothiophene) Organic Semiconductor Thin Films Involving Oxygen and Sorbed Moisture. *Adv. Mater.* **2009**, *21*, 4747–4752, doi:10.1002/adma.200901120.
6. Singh, A.K.; Pandey, A.; Chakrabarti, P. Fabrication, modelling and characterization of green light photosensitive p-channel - Poly[2,5-bis(3-tetradecylthiophen-2-yl)thieno[3,2- 784 b]thiophene] organic semiconductor based phototransistors. *Org. Electron.* **2019**, *75*, 105424, doi:10.1016/j.orgel.2019.105424.
7. Singh, A.K.; Pandey, A.; Chakrabarti, P. Poly[2,5-bis(3-tetradecylthiophen-2-yl) thieno [3,2-b] thiophene] organic polymer based-interdigitated channel enabled thin film transistor for detection of selective low ppm Ammonia sensing at 25 °C. *IEEE Sens. J.* **2020**, *20*, 4047–4055, doi:10.1109/JSEN.2019.2963269.
8. DeLongchamp, D.M.; Kline, R.J.; Fischer, D.A.; Richter, L.J.; Toney, M.F. Molecular characterization of organic electronic films. *Adv. Mater.* **2011**, *23*, 319–337, doi:10.1002/adma.201001760.
9. Nikhil; Pandey, R.K.; Sahu, P.K.; Singh, M.K.; Prakash, R. Fast grown self-assembled polythiophene/graphene oxide nanocomposite thin films at air–liquid interface with high mobility used in polymer thin film transistors. J. Mater. Chem. C 2018, 6, 9981–9989, doi:10.1039/C8TC02485E.
10. Yu, S. H.; Cho, J.; Sim, K.M.; Ha, J.U.; Chung, D.S. Morphology-driven high-performance polymer transistor-based ammonia gas sensor. *ACS Appl. Mater. Interfaces* **2016**, *8*, 6570–6576, doi:10.1021/acsami.6b00471.
11. Lide, D.R. *CRC Handbook of Chemistry and Physics*; CRC Press: Boca Raton, FL, USA, 2005.

12. Sharma, N.; Sharma, N.; Srinivasan, P.; Kumar, S.; Balaguru Rayappan, J.B.; Kailasam, K. Heptazine based organic framework as a chemiresistive sensor for ammonia detection at room temperature. *J. Mater. Chem. A* **2018**, *6*, 18389–18395, doi:10.1039/C8TA06937A.
13. Jeevitha, G.; Abhinayaa, R.; Mangalaraj, D.; Ponpandian, N.; Meena, P.; Mounasamy, V.; Madanagurusamy, S. Porous reduced graphene oxide (rGO)/$WO_3$ nanocomposites for the enhanced detection of $NH_3$ at room temperature. *Nanoscale Adv.* **2019**, *1*, 1799–1811, doi:10.1039/C9NA00048H.
14. Amadei, G.; Ross, B.M. The reactions of a series of terpenoids with $H_3O^+$, $NO^+$ and $O_2^+$ studied using selected ion flow tube mass spectrometry. Rapid Commun. *Mass Spectrom.* **2011**, *25*, 162–168, doi:10.1002/rcm.4845.
15. Lu, M.-Y.; Xie, Y.; Huang, H.; Zhao, Y.-L.; An, H.-T.; Zhang, X.; Li, J.-R. Purification of borneol from its isomeric mixture by using metal–organic frameworks. *Sep. Purif. Technol.* **2023**, *304*, 122213, doi:10.1016/j.seppur.2022.122213.
16. Williams, D.E.; Smyth, C.P. The Dielectric Relaxation of Dibromodichloromethane, Succinonitrile and Several Camphor Derivatives in the Solid State. *J. Am. Chem. Soc.* **1962**, *84*, 1808–1812, doi:10.1021/ja00869a007.


**Disclaimer/Publisher's Note:** The statements, opinions and data contained in all publications are solely those of the individual author(s) and contributor(s) and not of MDPI and/or the editor(s). MDPI and/or the editor(s) disclaim responsibility for any injury to people or property resulting from any ideas, methods, instructions or products referred to in the content.
